# Winding up the molecular clock in the genus *Carabus *(Coleoptera: Carabidae): assessment of methodological decisions on rate and node age estimation

**DOI:** 10.1186/1471-2148-12-40

**Published:** 2012-03-28

**Authors:** Carmelo Andújar, José Serrano, Jesús Gómez-Zurita

**Affiliations:** 1 Departamento de Zoología y Antropología Física. Facultad de Veterinaria, Universidad de Murcia, 30071 Murcia, Spain; 2 Institut de Biologia Evolutiva (CSIC-UPF), Pg. Marítim de la Barceloneta 37, 08003 Barcelona, Spain

**Keywords:** Molecular clock, Rates of molecular evolution, Deep node ages, Partitioning model, Clock model, Outgroup selection, Gblocks, Mitochondrial genes, Nuclear genes, Coleoptera, *Carabus*

## Abstract

**Background:**

Rates of molecular evolution are known to vary across taxa and among genes, and this requires rate calibration for each specific dataset based on external information. Calibration is sensitive to evolutionary model parameters, partitioning schemes and clock model. However, the way in which these and other analytical aspects affect both the rates and the resulting clade ages from calibrated phylogenies are not yet well understood. To investigate these aspects we have conducted calibration analyses for the genus *Carabus *(Coleoptera, Carabidae) on five mitochondrial and four nuclear DNA fragments with 7888 nt total length, testing different clock models and partitioning schemes to select the most suitable using Bayes Factors comparisons.

**Results:**

We used these data to investigate the effect of ambiguous character and outgroup inclusion on both the rates of molecular evolution and the TMRCA of *Carabus*. We found considerable variation in rates of molecular evolution depending on the fragment studied (ranging from 5.02% in *cob *to 0.26% divergence/My in *LSU-A*), but also on analytical conditions. Alternative choices of clock model, partitioning scheme, treatment of ambiguous characters, and outgroup inclusion resulted in rate increments ranging from 28% (*HUWE1*) to 1000% (*LSU-B *and *ITS2*) and increments in the TMRCA of *Carabus *ranging from 8.4% (*cox1-A*) to 540% (*ITS2*). Results support an origin of the genus *Carabus *during the Oligocene in the Eurasian continent followed by a Miocene differentiation that originated all main extant lineages.

**Conclusions:**

The combination of several genes is proposed as the best strategy to minimise both the idiosyncratic behaviors of individual markers and the effect of analytical aspects in rate and age estimations. Our results highlight the importance of estimating rates of molecular evolution for each specific dataset, selecting for optimal clock and partitioning models as well as other methodological issues potentially affecting rate estimation.

## Background

Time calibration of phylogenetic trees is a key factor to reconstruct the evolutionary history of taxa [1]. This is usually accomplished by extrapolating the known age of a node (e.g. based on fossil data) to the remainder of the tree, and assuming a molecular clock. Some animal groups, such as mammals or birds, are especially suited for time estimation based on a rather complete fossil record, but other organisms, typically species-rich groups of invertebrates with poor or inexistent fossil record, may represent more of a challenge for a similar exercise. In the case of insects, for instance, reliable paleontological evidence is frequently lacking, which leads to applying a proposed standard rate of 2.3% divergence/My for the insect mitochondrial genome [2]. However, the development of independent calibration analyses, mainly based on the age of geologic phenomena that underlie the origin of particular cladogenetic events, have found rates either slower e.g., [3-5] or faster e.g., [6-9] than this standard. These studies illustrate how often the routine application of a standard rate may lead to incorrect inference of evolutionary histories.

The discrepancies in the rates of molecular evolution can be attributed to lineage specific effects or to the molecular marker employed [1,10-12]. However, they could also reflect biases in the calibration procedure [13]. Decisions relative to the analytical procedure with potential effects on estimated rates include the suitability of selected lineage splits and the strategy used to enforce ages to nodes [14-16], methodological aspects such as the method for branch length estimation (i.e., maximum likelihood vs. Bayesian methods; [17,18]), model of among-branch rate variation (i.e., strict vs. relaxed clock models; [19-22]), selection of evolutionary model e.g., [9], partitioning of data e.g., [12,23], taxon sampling e.g., [24] or inclusion of ambiguously aligned regions [25]. But the effects of the methodology and specifically the combined effect of choices relative to these methodological aspects have not been fully explored. The increasing number of studies that rely on calibration analyses highlight the importance of investigating how these factors influence evolutionary rate and node age estimation in real datasets.

Within the Coleoptera, the family Carabidae has been the focus of several molecular clock calibration attempts. For instance, Contreras-Diaz et al. [7] and Ruiz et al. [5] estimated *cox1-cox2 *rates of 3.04% and 0.92% divergence/My for Canarian species of *Trechus *and Sphodrini ground beetles, respectively. Prüser and Mossakowski [3] used a strict global clock method and the opening of the Gibraltar Strait at the end of the Miocene to calibrate hypothetic vicariance events between Iberian and North African populations of *Carabus *species. They found rates between 0.39 and 0.98% divergence/My for *nd1 *data. Su et al. [26] and Tominaga et al. [27] investigated *nd5 *rates for two endemic subgenera of Japanese *Carabus *using a similar approach and the isolation of Japan from the continent at 15 Mya. These authors found a very low evolutionary rate for this gene, 0.28% divergence/My.

The genus *Carabus *is a Holarctic taxon that includes about 950 species, currently divided in eight main divisions [28]. It is highly diversified in the Palaearctic, where it is distributed throughout continental Eurasia, but is also present in Japan, Iceland, the Canary Islands, North Africa and several Mediterranean islands. Indeed, the genus *Carabus *represents a good research subject to conduct comparative calibration analyses, since there are a fair number of available DNA sequences, a relatively solid systematic knowledge of its limits and major splits [29], and the evolutionary history of these apterous beetles can be linked to geologic events that provide multiple potential calibration hypotheses about the origin of clades.

Here we perform calibration analyses on nine gene fragments that include protein coding and ribosomal genes belonging to both mitochondrial and nuclear genomes. Calibration analyses are based on the ages of the most recent common ancestor (TMRCA) for three cladogenetic events, including the origin of subgenera *Mesocarabus, Macrothorax *and the sister pair *Eurycarabus *and *Nesaeocarabus*, as estimated on an *nd5 *phylogeny using eight calibration points based on dates for fossil and past geologic events. The procedure takes into account the uncertainty intervals of these calibration points, to avoid a false perception of precision on age estimation in subsequent calibration analyses. Overall, we have conducted a total of 152 independent calibration analyses on individual and concatenated DNA matrices, using different outgroups, clock models, partition schemes and alternative treatments of ambiguous characters. We used these data to address several specific aims: (i) to obtain a reliable time scale for the origin and evolution of the genus *Carabus *and discuss the obtained rates of molecular evolution with those reported in other studies, and most critically (ii) to evaluate the effect of methodological decisions relative to calibration analyses in the resulting calibrated phylogenies.

## Methods

### Taxon and gene sampling

Thirty-four specimens belonging to the family Carabidae have been studied (Table 1). Samples correspond to 19 western Palearctic species of the genus *Carabus *representing 14 of the 91 conservatively recognized subgenera [28]. The selected species represent the eight main divisions and early splits in the phylogeny of *Carabus *as inferred from analyses based on two nuclear gene sequences [29], and hence the ingroup node in the present study very likely represents the true MRCA node of *Carabus*. Moreover, our sampling includes the taxa postulated as the earliest branching events in the evolution of the genus based on morphology, i.e. *Platycarabus, Rhabdotocarabus, Limnocarabus *and *Tachypus *[28,30]. Six taxa were incorporated as outgroups: *Calosoma *as sister group to *Carabus*; *Ceroglossus *and *Cychrus *as related members of the supertribe Carabitae; and *Leistus *and *Laemostenus *as more distantly related taxa. DNA was extracted from a leg of each specimen using the Dneasy Blood and Tissue kit (Qiagen, Hilden, Germany) or Invisorb Spin Tissue Mini Kit (Invitek, Berlin, Germany) following manufacturers' instructions.

**Table 1:** Species, locality data, voucher reference and accession numbers for each specimen and sequence.

Species	*Carabus *division	Locality	Voucher	*cox1*-A	*cox1*-B	*cob*	*rrnl*	*nd5*	*LSU*-A	*LSU*-B	*ITS2*	*HUWE1*
***Laemostenus terricola***	Outgroup	Alicante, Spain	TEUL4309	JQ693416	JQ689927	JQ689832	JF778796	JQ689864	JF778812	n/a	n/a	n/a
***Leistus spinibarbis***	Outgroup	Albacete, Spain	CMOJ3909	JQ693414	JQ689906	JQ689811	JF778797	n/a	JF778813	JQ689725	JQ689666	n/a
***Calosoma aeropunctatum***	Outgroup	Susuz, Turkey	TURQ52001	JQ689899	JQ689933	JQ689838	JQ689804	JQ689870	JQ689719	JQ689750	JQ689691	JQ689776
***Calosoma sycophanta***	Outgroup	Albacete, Spain	CALO25405	JQ693413	JQ689904	JQ689809	JF778798	JQ689842	JF778814	JQ689723	JQ689664	JQ689754
***Ceroglossus chilensis***	Outgroup	Chiloe, Chile	CHIL1-GA	JQ689875	JQ689905	JQ689810	JQ689779	JQ689843	JQ689696	JQ689724	JQ689665	n/a
***Cychrus semigranosus***	Outgroup	Pirin Mts., Bulgaria	CYCH1003	JQ689876	JQ689907	JQ689812	JQ689780	JQ689844	JQ689697	n/a	JQ689667	n/a
***C. (Archicarabus) nemoralis***	Archicarabomorphi	Navarra, Spain	RONC1549	JQ689889	JQ689921	JQ689826	JQ689793	JQ689858	JQ689709	JQ689739	JQ689680	n/a
***C. (Platycarabus) irregularis***	Arcifera	Resita, Rumania	ROMA94006	JQ689887	JQ689919	JQ689824	JQ689791	JQ689856	JQ689707	JQ689737	JQ689678	JQ689764
***C. (Rhabdotocarabus) melancholicus***	Arcifera	Cádiz, Spain	EALM42207	JQ689877	JQ689908	JQ689813	JQ689781	JQ689845	JQ689698	JQ689726	JQ689668	JQ689755
***C. (Rhabdotocarabus) melancholicus***	Arcifera	Toledo, Spain	ROBU37	JQ689885	JQ689917	JQ689822	JQ689789	JQ689854	n/a	JQ689735	JQ689676	JQ689762
***C. (Rhabdotocarabus) melancholicus***	Arcifera	Tidiquin, Morocco	TIDI39104	JQ689895	JQ689928	JQ689833	JQ689800	JQ689865	JQ689715	JQ689745	JQ689686	JQ689771
***C. (Carabus) deyrollei***	Digitulati	Lugo, Spain	GALI1553	JQ693415	JQ689911	JQ689816	JF778799	JQ689848	JF778815	JQ689729	n/a	n/a
***C. (Eurycarabus) famini***	Digitulati	Ketama, Morocco	MORR54807	JQ689884	JQ689916	JQ689821	JQ689788	JQ689853	JQ689705	JQ689734	JQ689675	JQ689761
***C. (Eurycarabus) famini***	Digitulati	El Alia, Tunisia	EURY1625	JQ689878	JQ689909	JQ689814	JQ689782	JQ689846	JQ689699	JQ689727	JQ689669	JQ689756
***C. (Nesaeocarabus) abbreviatus***	Digitulati	Tenerife, Spain	TENE15007	JQ689894	JQ689926	JQ689831	JQ689799	JQ689863	JQ689714	JQ689744	JQ689685	JQ689770
***C. (Nesaeocarabus) abbreviatus***	Digitulati	Tenerife, Spain	BABA44	JQ689874	JQ689903	JQ689808	JQ689797	n/a	JQ689695	n/a	JQ689663	n/a
***C. (Morphocarabus) monilis***	Lipastrimorphi	Drome, France	SAOU1538	JQ689890	JQ689922	JQ689827	JQ689794	JQ689859	JQ689710	JQ689740	JQ689681	JQ689766
***C. (Mesocarabus) dufourii***	Metacarabi	Cordoba, Spain	ZUHE111	JQ689902	JQ689936	JQ689841	JQ689807	JQ689873	JQ689722	JQ689753	JQ689694	JQ689778
***C. (Mesocarabus) lusitanicus***	Metacarabi	Ciudad Real, Spain	VESC5	JQ689901	JQ689935	JQ689840	JQ689806	JQ689872	JQ689721	JQ689752	JQ689693	JQ689777
***C. (Mesocarabus) macrocephalus***	Metacarabi	La Coruña, Spain	FORO157	JQ689879	JQ689910	JQ689815	JQ689783	JQ689847	JQ689700	JQ689728	JQ689670	JQ689757
***C. (Mesocarabus) riffensis***	Metacarabi	Ketama, Morocco	KETA569	JQ689881	JQ689913	JQ689818	JQ689785	JQ689850	JQ689702	JQ689731	JQ689672	JQ689758
***C. (Chrysocarabus) auronitens***	Neocarabi	Resita, Rumania	ROMA93906	JQ689886	JQ689918	JQ689823	JQ689790	JQ689855	JQ689706	JQ689736	JQ689677	JQ689763
***C. (Chrysocarabus) rutilans***	Neocarabi	Barcelona, Spain	SENY1548	JQ689891	JQ689923	JQ689828	JQ689795	JQ689860	JQ689711	JQ689741	JQ689682	JQ689767
***C. (Lamprostus) coriaceus***	Neocarabi	Oysu, Turkey	TURQ91306	JQ689900	JQ689934	JQ689839	JQ689805	JQ689871	JQ689720	JQ689751	JQ689692	n/a
***C. (Macrothorax) morbillosus***	Neocarabi	Murcia, Spain	MAZA5908	JQ689883	JQ689915	JQ689820	JQ689787	JQ689852	JQ689704	JQ689733	JQ689674	JQ689760
***C. (Macrothorax) morbillosus***	Neocarabi	Sejenane, Tunisia	TUNA1622	JQ689896	JQ689929	JQ689834	JQ689801	JQ689866	JQ689716	JQ689746	JQ689687	JQ689772
***C. (Macrothorax) morbillosus***	Neocarabi	Bazia, Tunisia	TUNB1623	JQ689897	JQ689930	JQ689835	JQ689802	JQ689867	JQ689717	JQ689747	JQ689688	JQ689773
***C. (Macrothorax) morbillosus***	Neocarabi	El Alia, Tunisia	TUNC1624	JQ689898	JQ689931	JQ689836	JQ689803	JQ689868	JQ689718	JQ689748	JQ689689	JQ689774
***C. (Macrothorax) rugosus***	Neocarabi	Ksar-el-Kebir, Morocco	KSAR65807	JQ689882	JQ689914	JQ689819	JQ689786	JQ689851	JQ689703	JQ689732	JQ689673	JQ689759
***C. (Macrothorax) rugosus***	Neocarabi	Cádiz, Spain	SMAR43907	JQ689892	JQ689924	JQ689829	JQ689796	JQ689861	JQ689712	JQ689742	JQ689683	JQ689768
***C. (Megodontus) violaceus***	Neocarabi	Popovi Livadi, Bulgaria	POPO83000	JQ689880	JQ689912	JQ689817	JQ689784	JQ689849	JQ689701	JQ689730	JQ689671	n/a
***C. (Limnocarabaus) clatratus***	Spinulati	Susuz, Turkey	TURQ51901	JQ693417	JQ689932	JQ689837	JF778800	JQ689869	JF778816	JQ689749	JQ689690	JQ689775
***C. (Tachypus) cancellatus***	Tachypogenici	France	TACHcc816	JQ689893	JQ689925	JQ689830	JQ689798	JQ689862	JQ689713	JQ689743	JQ689684	JQ689769
***C. (Tachypus) cancellatus***	Tachypogenici	Resita, Rumania	ROMA94106	JQ689888	JQ689920	JQ689825	JQ689792	JQ689857	JQ689708	JQ689738	JQ689679	JQ689765

Division of genus *Carabus *follows Deuve [28]

Each specimen was characterized for nine DNA fragments corresponding to seven different ribosomal and protein coding genes from mitochondrial (*cox1-A, cox1-B, nd5, cytb, rrnL*) and nuclear (*LSU-A, LSU-B, ITS2, HUWE1*) genomes (Additional file 1: Table S1). The sequences of *HUWE1 *are homologous to the Anonymous gene described by Sota and Vogler [31] for the genus *Carabus*, and to the predicted HUWE1 gene as identified by BLAST searches against the *Tribolium castaneum *genome. PCR reactions were made using PuReTaq Ready-To-Go PCR beads (GE Healthcare, UK) or Qiagen Taq Polymerase with 39 cycles at 50-54°C for primer annealing. Purification of PCR products and sequencing in both directions with the same primers used for PCR was performed by Macrogen Inc. (Seoul, Korea). Sequence accession numbers are given in Table 1.

### Sequence alignment

Mitochondrial protein coding genes were unambiguously aligned and checked for their correct translation to amino acids using Mega 4 [32]. The 5'-end of the *HUWE1 *fragment was also unambiguously aligned and correctly translated to amino acids, while the 3'-end showed a length-variable intron sequence. All ribosomal markers (*rrnL, LSU-A, LSU-B *and *ITS2*) also showed length variation and required objective alignment prior to phylogenetic analysis.

Variable-length DNA fragments for the dataset including outgroups were aligned under each combination of five iterative refinement methods (FFT-NS-i, E-INS-i, G-INS-I, L-INS-I and Q-INS-I) and three scoring matrices (1-PAM, 20-PAM and 200-PAM) in Mafft 6.240 [33,34]. Each individual alignment was assessed for congruence with respect to a combined matrix including unambiguously aligned regions of every gene. To get this combined matrix, every fragment was independently aligned in MAFFT with FFT-NS-i parameters, and local ambiguities were removed with Gblocks [35] with the *No-gaps* option and other default parameters; the resulting fragments were concatenated. Congruence was measured using both the incongruence length difference index (ILD; [36]) and the rescaled ILD [37]. ILD values were estimated from parsimony-based tree lengths in every case using PAUP* 4.0 [38]. The alignment conditions maximizing character congruence for every length-variable marker, i.e. producing the lowest rescaled ILD value, were objectively selected as those generating the best homology hypothesis for these data and employed in subsequent analyses.

Favored alignments were used to produce three concatenated matrices, including all mitochondrial fragments (MIT), all nuclear fragments (NUC) and both datasets (MIT-NUC). In the case of ribosomal genes, selected alignments were previously processed with Gblocks [35] using the *All-gaps *option and default parameters; only selected positions were included in the concatenated matrices. These alignments are available at Treebase.org (submission number 12410: http://purl.org/phylo/treebase/phylows/study/TB2:S12410)

### Phylogenetic analyses

All phylogenetic and calibration analyses described below were run independently for both the complete dataset (*outgroup *dataset) and a subset of data representing only the 28 ingroup *Carabus *sequences (*ingroup *dataset).

Bayesian phylogenetic inference for each individual and concatenated datasets, without specifying partitions and without clock assumptions, were run with MrBayes 3.1 [39,40] under a GTR + G + I model to assess the reliability of nodes to be used in subsequent calibration tests. The GTR model of nucleotide substitution was identified by jModeltest [41] as the best fitting for *nd5, cox1-B, LSU-A, LSU-B, ITS2 *and all concatenated datasets. For the remaining genes (*cox1-A, cob, rrnL *and *HUWE1*) a simpler model was favored, but the GTR was second best. The parameter gamma (G) was favored for all data sets but not invariant characters (I) in the case of nuclear ribosomal genes, although this parameter was included in the second best fitting model. To homogenize this part of the methodology, a complex GTR + G + I model was used in every analysis as their parameters are co-estimated with the tree and they can match eventually any simpler model at expense of increasing perhaps the variance of estimates. Analyses enforced two independent runs, each with three hot and one cold chain, for 20,000,000 generations, whereby trees were sampled every 1,000 generations. Convergence of independent runs was checked in Tracer 1.5 [42] and their half compatible consensus tree was calculated excluding 10% of initial trees, after the plateau in tree likelihood values had been reached. Trees were visualized using FigTree 1.1.2 [43] and node posterior probabilities were interpreted as support values.

### Calibration analyses

**Figure 1:**
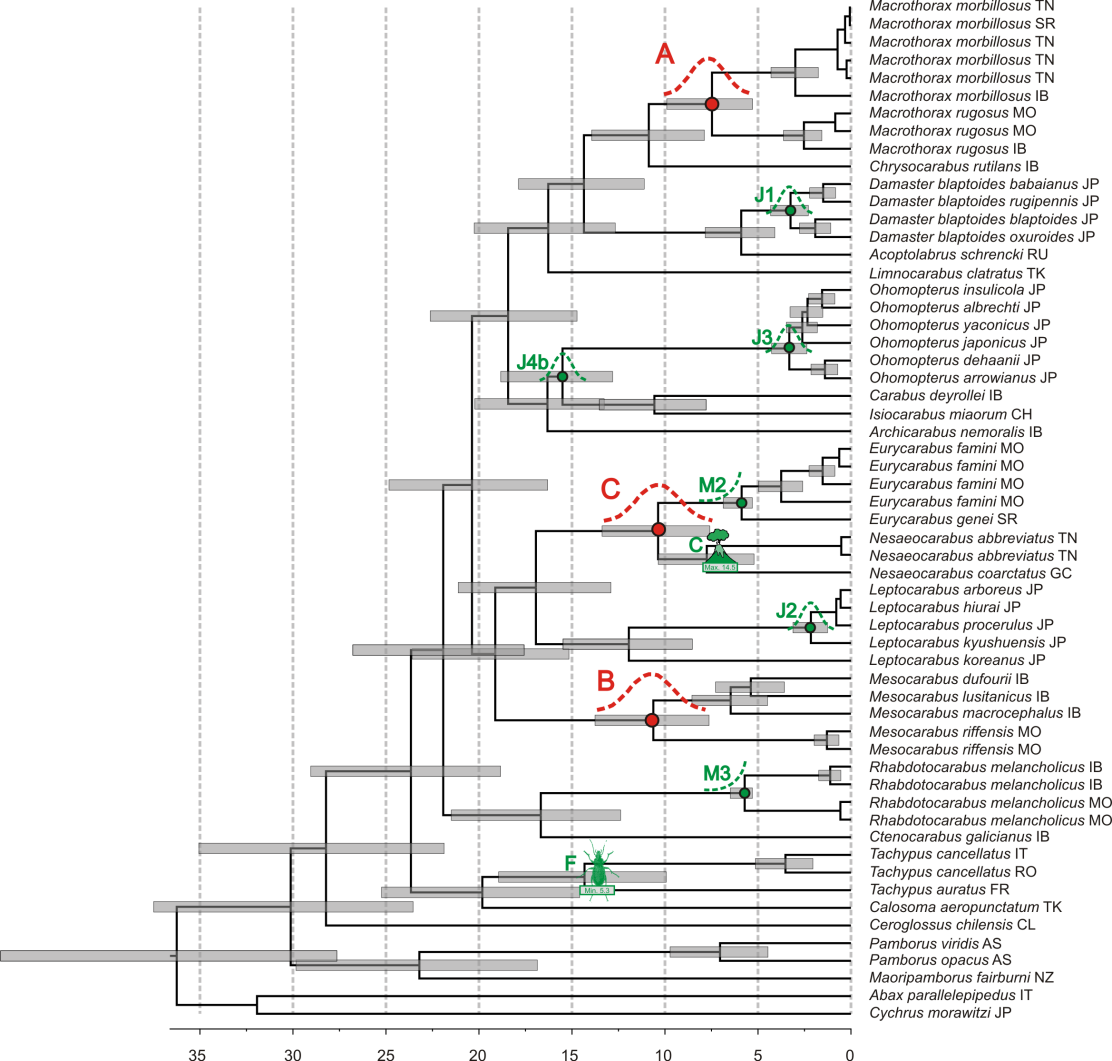
**Ultrametric time-calibrated phylogenetic tree obtained with BEAST for *nd5 *data in *Carabus***. Nodes J1, J2, J3, J4b, M2, M3, C and F (labeled green) were used as calibration points (detailed information on node age priors as shown in Table 2). The HPD distribution of ages obtained for nodes A, B and C (labeled red) are used as priors for subsequent calibration analyses on the different individual and combined datasets.

Calibration tests were standardized across the phylogenies estimated under different conditions, by using a fixed set of nodes constrained to absolute age intervals obtained from an independent analysis of an expanded *nd5 *dataset. This dataset included 51 *Carabus *and 7 outgroup *nd5 *sequences (Additional file 1: Table S2) and was analyzed in BEAST 1.5.4 [44] constraining eight calibrations points based on geologic events and the availability of a fossil *Carabus *from the end of the Miocene (Figure 1, Table 2). These data were investigated under different codon partition schemes (1P: no partitioning; 2P: two partitions, considering first and second codon positions together; 3P: each codon position as a different partition) and clock models, strict (SC) and uncorrelated lognormal (ULN) clocks. The optimal conditions were selected based on Bayes factors (BF) comparisons using marginal likelihood values as calculated in Tracer 1.5. Positive evidence based on BF was interpreted as requiring at least a ten units increase in marginal likelihood per additional free parameter before accepting a more complex model [45,46]. We assumed one extra parameter in ULN analyses compared to the SC assumption [20], and ten extra parameters per additional partition under a GTR + G + I model.

**Table 2:** Calibration hypotheses employed to time-calibrate the initial phylogeny of the genus *Carabus *and related taxa based on the *nd5 *gene

Evolutionary event: node	Calibration event	Event age (Ma)	Priors on node ages	Prior 95% age interval
Split between two Canarian endemic species: *Carabus (Nesaeocarabus) coarctatus* and *C. (N.) abbreviates*: **C**	Volcanic emergence of Gran Canaria	14.5	Uniform (*a *= 0, *b *= 14.5)	0.03-14.14

*Carabus (Autocarabus) cancellatus *fossil: **F**	Messinian deposits of Cantal (France)	5	Lognormal (*μ *= 25,*σ *= 1.5, offset = 5)	5.4-158.5

Radiation of *Damaster*: **J1**	Final disconnection of Japan from mainland	3.5	Truncated Normal (*μ *= 3.5,*σ *= 1, *a *= 0.1, *b *= 1000)	1.55-5.46

Radiation of *Leptocarabus*: **J2**	Final disconnection of Japan from mainland	3.5	Truncated Normal (*μ *= 3.5,*σ *= 1, *a *= 0.1, *b *= 1000)	1.55-5.46

Radiation of *Ohomopterus*: **J3**	Final disconnection of Japan from mainland	3.5	Truncated Normal (*μ *= 3.5,*σ *= 1, *a *= 0.1, *b *= 1000)	1.55-5.46

Split between subgenus *Isiocarabus *and *Ohomopterus*: **J4**	Initial disconnection of Japan from mainland	15	Normal (*μ *= 15,*σ *= 1)	13.04-16.96

Split between *Carabus (Eurycarabus) genei *from Corsica and North African *Eurycarabus*: **M2**	Opening Gibraltar strait	5.33	Exponential (*μ *= 0.5, offset = 5.3)	5.31-7.14

Split between two *Carabus (Rhabdotocarabus) melancholicus *subspecies: **M3**	Opening Gibraltar strait	5.33	Exponential (*μ *= 0.5, offset = 5.3)	5.31-7.14

The resulting calibrated phylogeny was used to obtain the ages for three well-supported cladogenetic events in the phylogeny of *Carabus: *node A, the split between *Carabus (Macrothorax) rugosus *and *C. (Macrothorax) morbillosus*; node B, the split of *Carabus (Mesocarabus) riffensis *from European *Mesocarabus*; and node C, the split between the sister subgenera *Eurycarabus *and *Nesaeocarabus*. These nodes were selected because they are not affected by systematic conflict (and they are generally retrieved in the phylogenies of each gene investigated), they are old enough to avoid time dependence effects [47] and not so deep as to be excessively affected by saturation of molecular change. We used TreeStat 1.6.1 [48] to recover these node ages from the sample of the MCMC search in BEAST and used the "fitdistr" option of the R package MASS to obtain a gamma function adjusting the distribution of sampled ages, thus including uncertainty associated to the age estimations (Table 3).

**Table 3:** Calibration points employed to time-calibrate molecular phylogenies of single and combined datasets in *Carabus*

NODE	CLADOGENETIC EVENT	AGE and 95% HPD interval (Ma)	GAMMA DISTRIBUTION
A	Split between *Carabus (Macrothorax) rugosus *and *C. (Macrothorax) morbillosus*	7.48 (6.05-9.14)	Shape: 63.787; Scale: 0.118

B	Split of *Carabus (Mesocarabus) riffensis *from European *Mesocarabu*	10.93 (8.90-13.26)	Shape: 68.490; Scale: 0.1604

**C**	Split between the subgenera *Eurycarabus *and *Nesaeocarabus*	9.51 (7.71-11.56)	Shape: 66.361; Scale: 0.144

Calibration analyses of each marker and their combination were conducted in BEAST 1.5.4 based upon four independent runs of 50 million generations each, sampling every 2,000th generation, and using a Yule tree prior and a GTR + G + I evolutionary model, with ten categories for the gamma distribution. Samples from these independent runs were compared, checked for convergence and combined after conservatively removing 10% of initial trees in Logcombiner 1.5.4 [44], drawing one sample every 8,000th generation. Mean, standard error, highest posterior density intervals (95%HPD) and effective sample size of likelihood, evolutionary rates and the TMRCA of *Carabus *were inspected using Tracer 1.5. Consensus trees were obtained in TreeAnnotator 1.5.4 [44] using the mean age option.

*Calibration analyses on individual protein coding genes*. Protein coding genes, including the 5'-end of the *HUWE1 *fragment, were analyzed under different clock assumptions including SC and ULN clocks, as well as different codon partition schemes (1P, 2P and 3P), for both the *outgroup *and *ingroup *datasets. Additionally, the complete *HUWE1 *gene fragment (i.e. including the non-coding 3'-end) was analyzed without codon partitioning. Results were analyzed to simultaneously select the best clock and partition model using BF as above.

*Calibration analyses on individual ribosomal genes*. Calibration analyses for trees based on ribosomal genes were conducted considering (i) all positions (*complete *dataset), (ii) only positions selected by Gblocks with the *No-gaps *option and other default parameters (*nogaps *dataset), and (iii) only positions selected with the *All-gaps *option (*allgaps *dataset). Each individual analysis was done under different clock assumptions (SC and ULN) and for the *outgroup *and *ingroup *matrices. The best clock model was assessed in every case using BF comparisons as above.

*Calibration analyses on concatenated datasets. Outgroup *and *ingroup *datasets for the MIT, NUC and MIT-NUC concatenated matrices were analyzed in BEAST under different clock assumptions (SC and ULN) following different partition schemes, which included no data partitioning (NP), partitioning by gene but not by codon in the case of protein coding genes (G-1P), partitioning by gene, and each protein coding gene with two codon partitions where first and second positions are considered together (G-2P), and partitioning by gene and by codon position (G-3P). Among-branch rate variation was always treated as linked among partitions, and BF comparisons were used again to select for the best clock and partitioning models.

The effect of different treatments of data was explored using Wilcoxon signed rank test on the values of two relevant parameters, including the estimated rate of molecular evolution for the marker or markers investigated and the estimated age of the ingroup node (TMRCA of *Carabus*).

## Results

### Phylogenetic framework for calibration tests

The alignment of choice based on congruence optimization with unambiguously aligned data was that obtained implementing the Q-INS-i algorithm for all nuclear genes, except for the *LSU-A *fragment in the case of the *ingroup *dataset. The latter, together with the mitochondrial ribosomal gene *rrnl *data, were optimal using the E-INS-i method (Additional file 1: Table S3).

Calibration analyses on the expanded *nd5 *gene dataset used a strict clock and 2P codon partitioning as selected by BF, resulting in a rate of molecular evolution of 0.0154 (95% HPD 0.0112-0.0198) substitutions per site per million years per lineage (subs/s/Ma/l). The time calibrated phylogeny obtained is shown in Figure 1. Nodes A, B and C were recovered with high support (posterior probability of 1.0) with median ages ranging from 7.5 Ma (node A) to 11.9 Ma (node B; Table 3).

**Figure 2:**
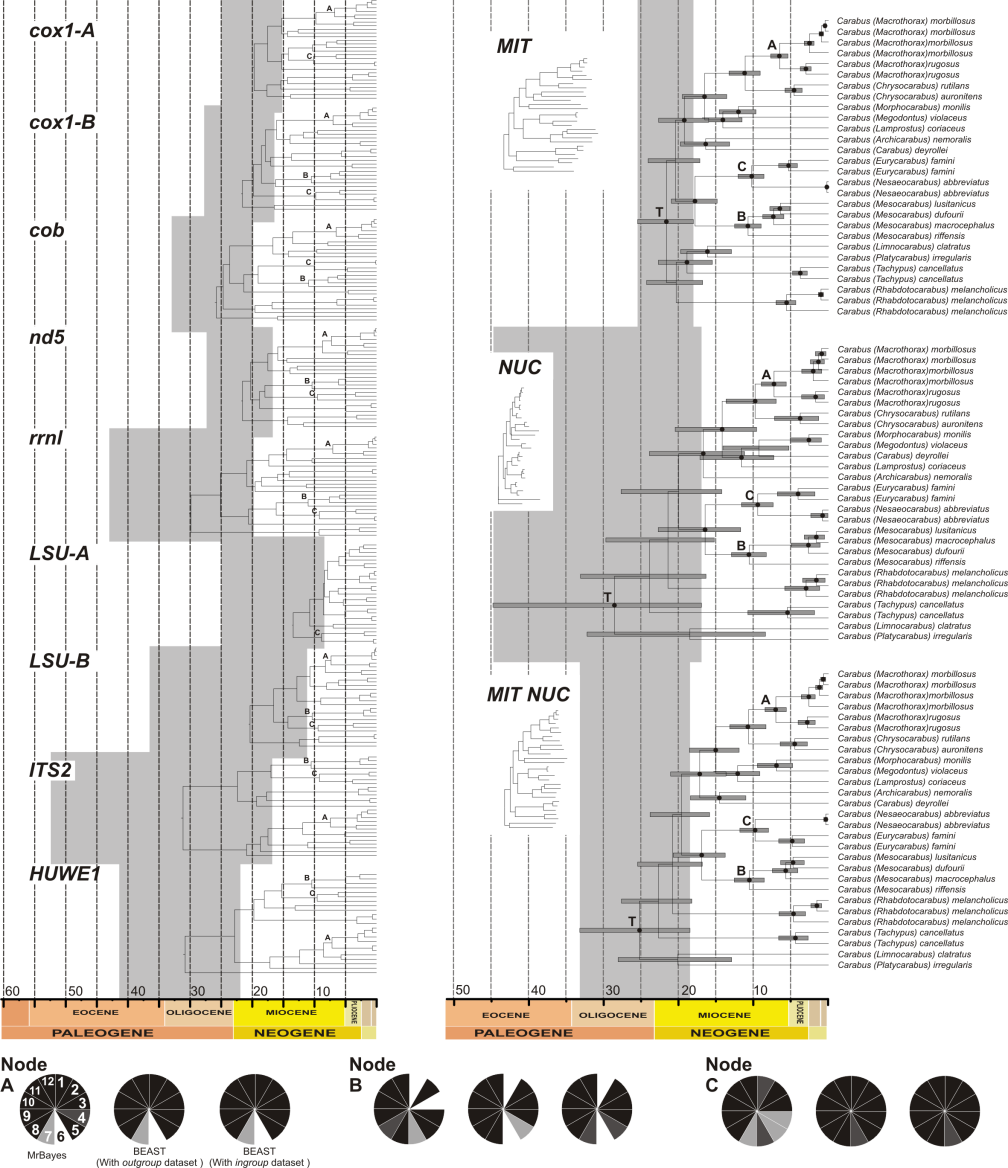
**Ultrametric time-calibrated trees obtained with BEAST for each individual (left) and combined datasets (right) of *Carabus***. Trees obtained with the *ingroup *dataset (including only *Carabus *species) and locus-specific optimal parameters. Nodes represented by black-filled circles received posterior probabilities (pp) higher than 0.9, bars represent 95% HPD intervals for node ages in Ma. The 95% HPD intervals of the TMRCA of *Carabus *are shaded in grey. Pie charts represent support (black, pp ≥ 0.95; dark grey, pp ≥ 0.85; light grey, pp ≥ 0.50; white, node not recovered) in MrBayes (*outgroup *dataset) and BEAST (*outgroup *and i*ngroup *datasets) for calibration nodes A, B and C, for the different gene fragments: *cox1-A *(1), *cox1-B *(2), *cob *(3), *nd5 *(4), *rrnL *(5), *LSU-A *(6), *LSU-B *(7), *ITS2 *(8), *HUWE1 *(9), MIT (10), NUC (11) and MIT-NUC (12).

For the mitochondrial dataset only two of the 34 specimens lacked one of the five sequenced fragments. In the case of nuclear genes six specimens lacked a single gene fragment, three lacked two loci and only one, within the outgroup taxa, lacked three fragments. Thus, completeness of the data matrices is very high and the effect of missing data is expected to be low. Bayesian phylogenetic analyses for the concatenated datasets resulted in the recovery of focal nodes A, B and C with posterior probability of 1.0 in all instances. Node A--split between *Carabus (Macrothorax) rugosus *and *C. (M.) morbillosus*--was found with a posterior probability (pp) higher than 0.95 for all individual DNA fragments except for *LSU-B *(pp = 0.74) and *LSU-A *(pp < 0.5). Node B--split of *Carabus (Mesocarabus) riffensis *from European *Mesocarabus*--appeared highly supported (pp > 0.95) for most individual DNA fragments, except for *ITS2 *(pp = 0.9), *LSU-A *(pp = 0.67), *cox1-A *and *cob *(pp < 0.5). Finally, node C--split between the subgenera *Eurycarabus *and *Nesaeocarabus*--was recovered for all individual fragments, although with pp < 0.85 in *nd5, rrnl *and *LSU-B*. Figure 2 shows support of nodes A, B and C as obtained with MrBayes and BEAST analyses.

**Figure 3:**
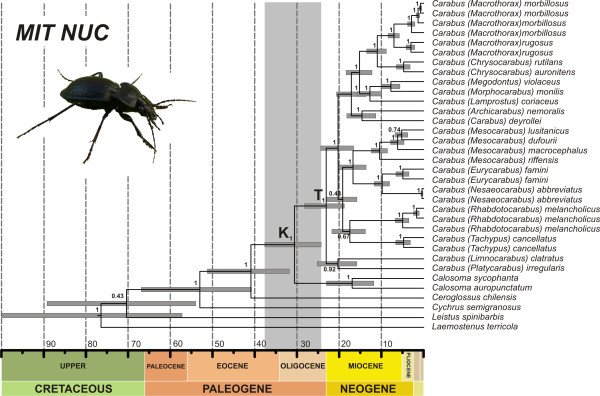
**Ultrametric time-calibrated tree for combined DNA markers (MIT-NUC dataset) of Carabidae**. Analyses were conducted in BEAST including outgroups, partitioning by gene, with two partitions for coding genes (first and second codon positions together) and applying a relaxed ULN clock. Node support is given as Bayesian posterior probabilities. Grey bars on nodes represent the 95% confidence intervals for node ages in Ma. The vertical grey bar shows the 95% HPD interval for the split between *Carabus *and *Calosoma*.

Analysis of individual genes frequently failed to recover the monophyly of the genus *Carabus *(node T) and/or its sister relationship with the genus *Calosoma *(node K) (Additional file 1: Figure S1, S2, S3, S4, S5, S6, S7, S8 and S9). However, these nodes were recovered with high support in all combined datasets (Figures 2, 3). Bayesian analyses conducted in BEAST, where the calibration age prior was applied together with the favored partition and clock scheme, slightly improved node support within the *Carabus *clade.

### Selection of optimal analytical conditions

For each individual and combined dataset and alternative partition and clock model schemes, the four independent BEAST runs resulted in similar global rates, TMRCA of *Carabus *and likelihood values, reaching stationary equilibrium and ESS values always higher than 500 for the likelihood parameter and higher than 200 for all the other parameters, with very few exceptions. The results from these independent runs and for each dataset were thus pooled together to generate samples of 180 million generations, with ESS values always higher than 200.

BF comparisons resulted in the selection of the 2P codon partition strategy for mitochondrial coding genes, the combination of all mtDNA markers, and the combination of all markers, while no data partitioning was selected for the nuclear *HUWE1 *fragment alone and for all nuclear markers combined (Additional file 1: Table S4). The strict clock was favored for all protein coding genes (including the entire *HUWE1 *gene fragment), the combined mtDNA including outgroup taxa, and also for some ribosomal genes using the *ingroup *dataset and after gap exclusion. Otherwise, the relaxed (ULN) clock was preferred for all other individual and combined markers (Additional file 1: Table S4). Relative to the effect of ambiguously aligned characters or outgroup inclusion/exclusion, no direct BF comparisons were possible, and a pragmatic decision was taken in every case. *Nogaps *datasets were discarded due to their major effect on rates and age estimations (see below). *Allgaps *and *complete *matrices showed similar rates and ages, and the first option was selected. Finally, we discarded outgroups for estimation of molecular rates and TMRCA of *Carabus *since this genus was not found monophyletic in most individual marker analyses, producing a net overestimation of the age for this node, taken as the one including all ingroup taxa but not only. When monophyly was recovered, such as in concatenated datasets, the differences in both rates and ages between *outgroup *and *ingroup *datasets was low.

### Evolutionary rates and ingroup ages

Table 4 shows the estimated rates of molecular evolution and the TMRCA of *Carabus *for each gene and their combinations, for the *ingroup *datasets and treated under optimal partitioning and clock assumptions. Rates of mitochondrial genes ranged from 0.0016 (95% HPD 0.0010-0.0022) substitutions per site per million years per lineage (subs/s/Ma/l) for the *rrnL *fragment, to 0.0251 (0.0151-0.0369) subs/s/Ma/l for the *cob *fragment. Important differences were also found for the estimated rates of nuclear genes, from 0.0013 (0.0007-0.0020) for the *LSU-A *gene fragment to 0.0064 (0.0037-0.0094) subs/s/Ma/l for *LSU-B*. As with *LSU*, the other gene characterized by two non-overlapping fragments (i.e., *cox1*) showed some differences despite using identical approaches: the 3'-end fragment (*cox1-B*) showed a faster rate of evolution, 0.0145 (0.0100-0.0198), than the *cox1-A *fragment, the one generally used as barcode, 0.0113 (0.0081-0.0147) subs/s/Ma/l. The rate obtained for the concatenation of all mitochondrial fragments was 0.0134 (0.0108-0.0162) subs/s/Ma/l, roughly equivalent to a divergence rate of 2.68% per Ma. The combination of nuclear fragments resulted in a rate of 0.0029 (0.0020-0.0039) subs/s/Ma/l (i.e., 0.58% per Ma).

**Table 4:** Rate of molecular evolution and TMRCA of *Carabus *for each individual fragment and combined datasets under optimal analytical conditions

Gene	Partition	Clock	Ambiguities treatment	Rate	TMRCA *Carabus*
***nd5***	NP*	SC	-	0.0159 (0.0102-0.0223)	20.71 (15.9-26.15)

***cox1-A***	2P	SC	-	0.0113 (0.0081-0.0147)	19.79 (15.2-24.9)

***cox1-B***	2P	SC	-	0.0145 (0.01-0.0198)	21.58 (16.43-27.71)

***cob***	2P	SC	-	0.0251 (0.0151-0.0369)	25.77 (19.75-32.91)

***rrnL***	NP	SC	allgaps**	0.0016 (0.001-0.0022)	29.91 (19.4-42.76)

***LSU-A***	NP	ULN	*allgaps*	0.0013 (0.0007-0.002)	13.37 (8.35-24.85)

***LSU-B***	NP	ULN	*allgaps*	0.0064 (0.0037-0.0094)	20.36 (11.23-36.61)

***ITS2***	NP	ULN	*allgaps*	0.0057 (0.0035-0.0081)	31.17 (16.8-52.47)

***HUWE1***	NP	SC	*complete*	0.0021 (0.0015-0.0027)	30.83 (21.79-41.36)

**MIT**	G-2P	SC	*allgaps*	0.0134 (0.0108-0.0162)	21.58 (17.98-25.4)

**NUC**	NP	ULN	*allgaps*	0.0029 (0.002-0.0039)	28.5 (16.97-44.65)

**MIT-NUC**	G-2P	ULN	*allgaps*	0.0080 (0.0064-0.0097)	25.16 (18.41-33.04)

Data corresponding to the *ingroup *dataset. Rates are given in substitutions per site per million years per lineage, and TMRCA of *Carabus *in millions of years before present (mean and 95% HPD interval)

* Selection of NP dataset due to the anomalous branch lengths resulting from codon partitioned *nd5 *analyses

** These rates are estimated after character culling in G-blocks and are necessarily an underestimation of the real rates of change accumulation

The mean estimated age of the ingroup oscillated between 13.4 (8.35-24.85) Ma as estimated for *LSU-A *data, and 31.2 (16.8-52.47) Ma, in the case of *ITS2*, whereas for most genes this value was between 20 and 30 Ma with widely overlapping 95% HPD intervals (Figure 2, Table 4). The combined analyses of all genes resulted in a TMRCA of *Carabus *of 25.2 (18.41-33.04) Ma. The dispersion of values around the mean was higher for genes analyzed under a relaxed clock. The split between *Carabus *and *Calosoma *(node K) was estimated to have occurred at around 30.6 Ma (95% HPD 24.27-37.65) with combined genes and the dataset including outgroup taxa (Figure 3).

### Effect of partitioning scheme

**Figure 4:**
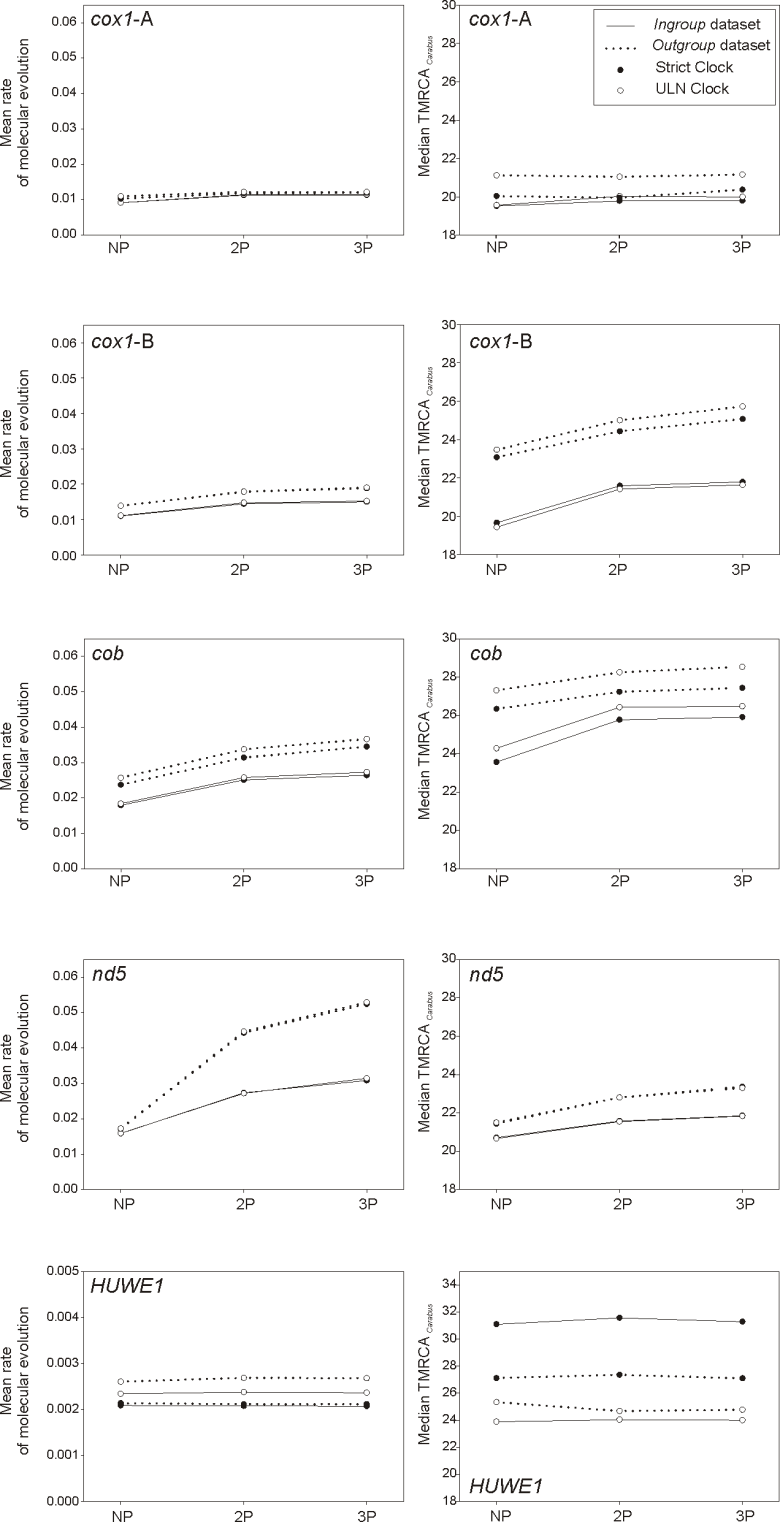
**Mean rates of molecular evolution and TMRCA of *Carabus *based on protein coding genes**. Rates are given in substitutions per site per million years per lineage, and TMRCA of *Carabus *in millions of years before present. Different partitioning schemes, clock models and outgroup inclusion/exclusion were considered.

**Figure 5:**
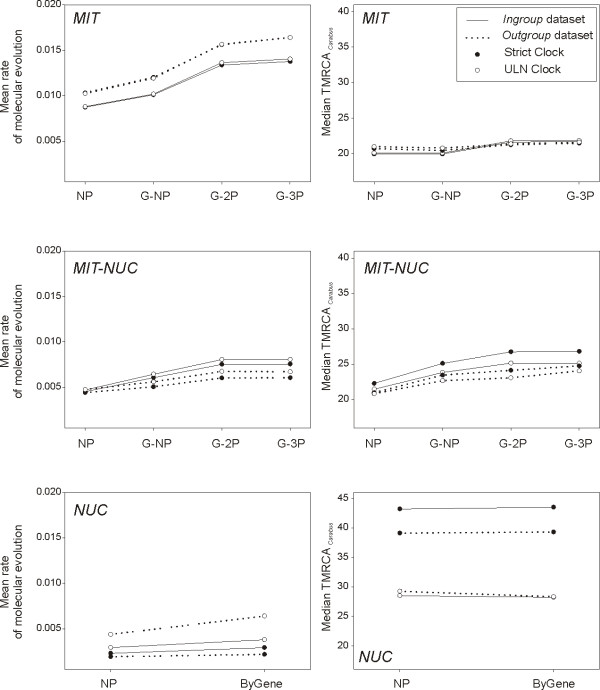
**Mean rates of molecular evolution and TMRCA of *Carabus *based on combined data**. Rates are given in substitutions per site per million years per lineage, and TMRCA of *Carabus *in millions of years before present. Different partitioning schemes, clock models and outgroup inclusion/exclusion were considered.

Data partitioning affected protein coding gene fragments (partitioning by codon positions) and concatenated datasets (partitioning by gene and by codon positions). A general trend was observed whereby the average values for the evolutionary rates and the estimated ingroup age increased with the number of partitions considered, both for individual markers (Wilcoxon signed rank test on mean rate, *P *< 0.01 for all partitioning treatment comparisons; Wilcoxon signed rank test on median TMRCA of *Carabus, P *< 0.01 for all comparisons) and their concatenation (Wilcoxon signed rank test on mean rate: *P *< 0.01 for all comparisons except for comparison of partitioning G-2P vs. G-3P, *P *= 0.058; Wilcoxon signed rank test on median TMRCA of *Carabus*: comparison of NP vs. G-1P, *P *= 0.151; G-1P vs. G-2P, *P *< 0.01; G-2P vs. G-3P, *P *= 0.016). This trend was found irrespective of the clock model enforced (Figures 4 and 5), and it was particularly exacerbated in the case of the rate estimated for *nd5 *and the entire dataset, which tripled its value compared to non-partitioned data when three partitions where considered, without remarkable effects on the estimation of the ingroup age. On the other hand, the coding region of the *HUWE1 *gene fragment showed invariable rates and estimated ingroup age independently of the partitioning strategy employed. Values for mean rates and TMRCA of *Carabus *and their associated 95% HPD intervals for each analysis are provided in Additional file 1: Tables S4 and S5, respectively.

### Effect of ambiguously aligned characters

**Figure 6:**
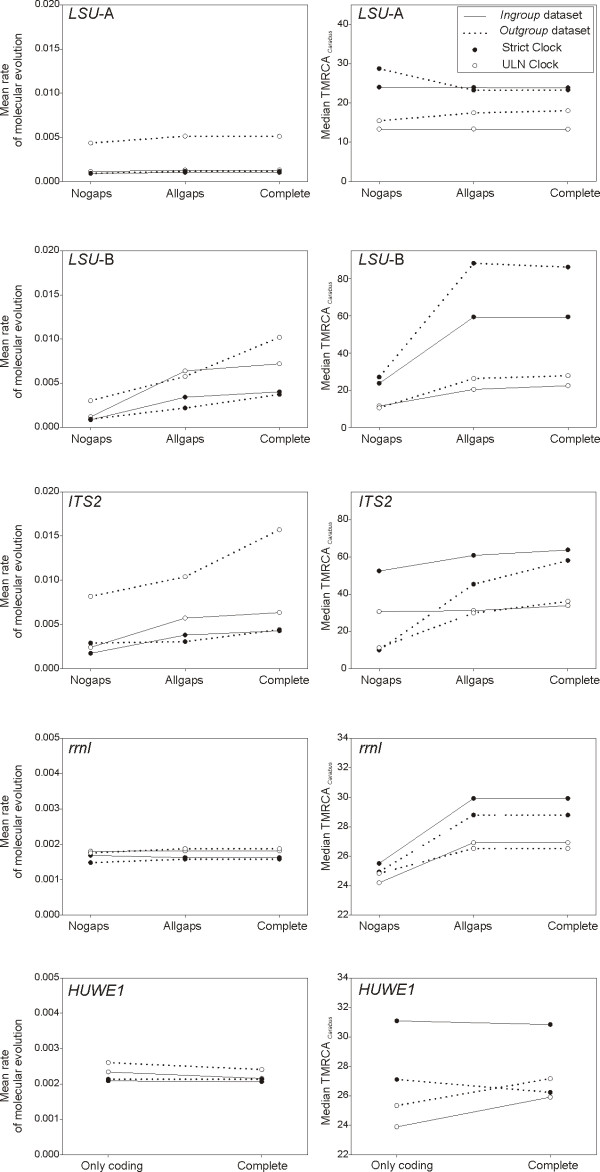
**Mean rates of molecular evolution and TMRCA of *Carabus *based on non-coding gene fragments**. Rates are given in substitutions per site per million years per lineage, and TMRCA of *Carabus *in millions of years before present. Different clock models, ambiguous character and outgroup inclusion/exclusion were considered.

The effect of gapped characters on rate and node age estimation was assessed in all gene fragments showing sequence length variation (Figure 6). In the case of ribosomal genes, the exclusion of gapped positions (*nogaps *option in Gblocks) had a noticeable effect lowering the estimates of evolutionary rates and ingroup age, up to three-fold, for the most variable gene fragments, hence with gappier alignments (*ITS2 *and *LSU-B*). These gene fragments diminished by 50 and 90% of aligned nucleotide positions, respectively, with a dramatic loss of phylogenetic information and great oscillations in the estimated parameters. Expectedly, more length-conserved fragments (*LSU-A *and *rrnL*) were only slightly affected by character culling in Gblocks, with 27 and 1% of character loss, respectively, under the most conservative *nogaps *treatment, and consequently showed lower variation, particularly in estimation of evolutionary rates. Less restrictive character culling approaches (*allgaps *option in Gblocks) preserved more characters to be used for branch length estimation and produced intermediate results, still with significant effects on rate estimation for highly variable markers, but not so much for that of the TMRCA of *Carabus *in the case of *ITS2 *and *LSU-B *data. The estimated mean node age in these cases was affected to a higher extent by outgroup inclusion/exclusion and by the clock model.

The simultaneous analysis of non-coding sequence information with exon information for the nuclear *HUWE1 *gene had little effect on the estimation of evolutionary rates, although the estimation of the ingroup age decreased or increased when assuming strict or relaxed clocks, respectively (Figure 6).

### Effect of clock model

The choice of strict versus relaxed clock had effects on the estimation of parameters of interest, generally associated with the actual clock model best fitting the data. Individual and combined genes in which the strict clock was preferred showed null to low effect of clock model on the estimation of rates and ingroup ages, with both parameters showing at most a trend to slightly higher values when a relaxed clock was enforced (Wilcoxon signed rank test on mean rate, *P *< 0.01; Wilcoxon signed rank test on median TMRCA of *Carabus, P *< 0.01). In all other cases, except for the total evidence dataset, the use of the suboptimal strict clock resulted in lower rate estimates and higher ingroup ages (Figures 4, 6, 5).

### Effect of outgroup

The dataset including outgroups generally resulted in higher rates of molecular evolution compared with the analyses using ingroup data only (Wilcoxon signed rank test, *P *< 0.01) (Figures 4, 6, 5). Exceptions to this pattern affected the fast evolving ribosomal markers *LSU-B *and *ITS2*, as well as the combination of nuclear genes and the total evidence dataset when investigated under a strict clock model. In turn, for the TMRCA of *Carabus *no general trend could be identified (Wilcoxon signed rank test, *P *= 0.1143), despite it was generally retrieved as older for most treatments when including outgroups, for instance with individual mitochondrial genes (Wilcoxon signed rank test, *P *< 0.01), a fact associated to most individual gene fragments failing to recover the monophyly of *Carabus*. Exceptionally, this age was younger for all nuclear markers independently (except *LSU-B*) and for their combination (NUC dataset) when the unfavored strict clock was enforced.

## Discussion

### A time scale for the origin and evolution of *Carabus*

The analyses of MIT, NUC and MIT-NUC combined datasets produce highly congruent topologies and high support for most nodes, including nodes T and K, representing the monophyly of *Carabus *and its sister relationship with *Calosoma*, respectively (Figure 3). In addition, all combined datasets show nearly complete overlap of the 95% HPD intervals on the estimated ages for these nodes; only the NUC dataset departs slightly from these values producing an older mean age estimate (Figure 2). The time scale obtained when all nuclear and mitochondrial genes are analyzed together (MIT-NUC dataset) situates the initial split between *Carabus *and *Calosoma *during the Oligocene, some 34 and 23 Ma, after the opening of the Atlantic Ocean and the split of the Nearctic and Palearctic regions [49]. This timing is congruent with the observation that *Carabus*, essentially a flightless genus, is more diverse in the Palaearctic (more than 900 species) than in the Nearctic region (12 species), whereas *Calosoma *is slightly more diverse in the Nearctic (ca. 90 species) than in the Palearctic region (76 species). The evolutionary events that originated the main extant lineages of *Carabus *took place according to our data during the early Miocene, between 23 and 16 Ma (Figure 3).

These findings disagree with the previous hypothesis assigning an older Eocene origin to *Carabus *[50]. The occurrence of North American endemic subgenera *Tanaocarabus *and *Lichnocarabus *was interpreted as evidence suggesting that the origin of the genus predated the opening of the Atlantic Ocean, considered the event responsible for the isolation of Nearctic from Western Palearctic lineages of *Carabus*. However, molecular data indicate that these Nearctic subgenera are instead related to Eastern Palearctic species [51,52], which suggests an origin for the genus *Carabus *within the Paleartic region and a more recent dispersal event across Bering Strait land bridges, in agreement with the dates here provided.

### Rates of molecular evolution in *Carabus*

The evolutionary rates for the genus *Carabus *as estimated here are, in general terms, higher than those reported in previous studies. The discrepancies are thought to be mostly related to the use of inappropriate calibration points in these studies, combined with simplistic corrections of genetic distances among species. Prüser and Mossakowski [3] in a study based on the *nd1 *gene in western Mediterranean species of the genus *Carabus*, calibrated the separation of six pairs of taxa with the opening of the Gibraltar Strait at the end of the Messinian (5.3 Ma). Five of these splits represented the separation of North African and European subspecies in *C. (Macrothorax) morbillosus *and *C. (Macrothorax) rugosus*, and the resulting rates ranged between 0.0020 to 0.0033 subs/s/Ma/l. However, these splits seemingly occurred well after the opening of the Gibraltar Strait (Andújar et al., unpublished data). The sixth node represented the split of subspecies of *Carabus (Rhabdotocarabus) melancholicus *from the Iberian Peninsula and North Africa, the only one with compelling evidence to represent a vicariant event resulting from the opening of Gibraltar Strait (Andújar et al., unpublished data). The rate estimated by Prüser and Mossakowski [3] was 0.0049 subs/s/Ma/l, much lower than our estimated rate for the slowest protein-coding gene--0.0113 (0.0081-0.0147) subs/s/Ma/l. Indeed, the divergence estimated by Prüser and Mossakowski [3] for these taxa, based on an uncorrected p-distance, was approximately half as low as distances corrected with appropriate evolutionary models (e.g., GTR + G model of evolution; [9]).

The degree of relaxation of evolutionary constraints acting on different genes or their portions ultimately reflects in differences on their inferred rate of molecular evolution [1,53]. Our data showed a 16-fold difference between the slowest (*rrnL*) and the fastest (*cob*) evolving mitochondrial markers, in agreement with rate variation estimates between particular mitochondrial genes found by Pons et al. [12] for Coleoptera. This important disparity cautions against the extrapolation of rates of molecular evolution among different gene fragments or their combinations. On the other hand, the extrapolation of evolutionary rates for the same marker across taxa, at least when they are relatively closely related, appears as a safer assumption, although it is possible to find differences. Several studies using a similar approach as that described here but targeting different families (and suborders) of Coleoptera produced slightly different rates of evolution for the same gene fragments. Thus, for instance, Papadopoulou et al. [9] and Ribera et al. [8] estimated rates of 3.54% and 4.08% divergence/Ma in Tenebrionidae and Leiodidae, respectively, for a fragment homologous to the *cox1-B *fragment investigated here, which in our case yielded a slower rate of 2.90% divergence/Ma (0.0145 subs/s/Ma/l; 95% HPD:0.01-0.0198) in *Carabus*. Evolutionary rates obtained by Pons et al. [12] for the *nd5 *(0.0168; 95% HPD 0.0086-0.0279) and *cob *(0.0172; 95% HPD 0.0071-0.0311) genes are very similar to those we obtained for *Carabus *(Table 4), while they found an unexpected higher rate for the *cox1 *gene (0.0861), although their estimation appeared associated to a surprisingly wide HPD interval (95% HPD 0.0251-0.1760).

### Heterogeneity in rates of molecular evolution

Invertebrate mitochondrial genomes have long been assumed to evolve at a standard molecular clock rate of 2.3% divergence/Ma [2]. This rate was deduced from heterogeneous mitochondrial data, including restriction fragment length polymorphism, DNA-DNA hybridization and sequence data for several genes. Its utilization in phylogenetic studies has been frequent for datasets composed by individual and concatenated mitochondrial DNA gene fragments (e.g., only in the case of beetles and for concatenated data: [7,54-61]). It is important to notice that in these studies similar rates for different taxa and different regions of the mitochondrial genome were assumed. This assumption has been lately refuted e.g., [9,11,12]. Moreover, in former studies where the standard rate was routinely applied the effects of methodological decisions on the calibration procedure had not been considered, a question that has been shown to be important [18,23], as we also stress here.

There is fair variation in the rates of individual gene fragments in *Carabus *which are affected by methodological aspects of the calibration procedure. Major differences on both the rates and the TMRCA of *Carabus *are obtained in the case of the nuclear ribosomal genes, which reach a fourfold variation between analyses involving different clock models, as well as inclusion/exclusion of ambiguous characters and/or outgroups. Mitochondrial genes show comparatively less variation due to methodological decisions, and only the *nd5 *fragment showed a twofold difference depending on treatment (but see below). The most remarkable observation is that a calibration based on combined datasets results in the lowest effect of prior analytical decisions on both evolutionary rate and node age estimation, together with a reduction of 95% HPD intervals associated to these estimates. This effect hints at the importance of using multiple gene data on calibration exercises, both as a way to average across genes, diluting idiosyncratic behaviors of stand-alone markers, but also to help the analyses to converge into more precise estimates.

### Clock calibration with protein coding genes

The response to changes in analytical conditions differs between protein coding mitochondrial markers and the *HUWE1 *gene fragment. Thus, for the latter, the selection of a suboptimal clock model produces marked differences in the estimation of both the rates of molecular evolution and the inferred TMRCA of *Carabus*, while this choice reveals no remarkable effects in the case of the mitochondrial genes. This behavior can be related to the underlying rate heterogeneity for each marker class: clock-like in the case of mtDNA data, but moderate in that of *HUWE1*. The selection of an unsuitable strict clock for the nuclear gene distorts branch length estimation and all parameters derived from these. In turn, the protein coding nuclear marker shows no apparent changes in the two parameters of interest through different partition schemes, while the mitochondrial genes prove more sensitive to complex evolutionary models, including the effect of adding or excluding an outgroup to the estimations. As with clock model selection, the partitioning scheme employed affects branch length estimation, and consequently the resulting inferred rate, a behavior already reported in other studies [23,62]. Mitochondrial protein coding genes show a trend to slightly higher inferred evolutionary rates and TMRCA of *Carabus *with increasing partitioning; and the same is true for the MIT and MIT-NUC datasets. The influence of codon site-specific models is similar to that found by Papadopoulou et al. [9], in their study of darkling beetles from the Aegean islands based on two mitochondrial and two nuclear fragments. These authors found up to 11% discrepancy in inferred rates between NP and 3P partitioning, higher for the latter, both under ULN clock assumption and using relatively recent calibration nodes (9-12 Ma), as used in our study. Conversely, other studies found the opposite trend, with younger estimated ages when using complex codon partitioning models for mitochondrial data (e.g., Nearctic and eastern Palaearctic skinks [23]). This trend can be caused by the same underlying problem related to the specific way in which saturation effects are corrected, but combined in this case with the use of deep calibration points (96-148 Ma) to infer younger node ages.

There seems to be an effect of the relative position of calibrating nodes on the ages extrapolated along the tree. Saturation and incorrect branch length estimation in deeper parts of the tree despite complex model-based corrections could explain these differences. The use of relatively recent calibration nodes should produce reliable age estimates for other nodes in areas of the tree not affected by saturation, with error accumulating progressively for deeper nodes, possibly with underestimated branch lengths, and in this case providing with minimum age estimates. Depth constraints restrain branch stretching in most of the tree, and when deep nodes are affected by incorrect length estimation due to saturation problems, errors in resulting node ages will be extrapolated to more recent parts of the tree, resulting in older than true time estimates for such recent nodes [63].

Bayesian phylogenetic inference is known to produce incorrect long branch estimates at least in the case mitochondrial codon-partitioned datasets [17,64]. These flawed estimations have been found to be stable across independent runs with fixed parameters, but also when Bayesian prior parameters are modified, an additional difficulty to detect them. It appears to be the case of the observed high rate increments when partitioning *nd5 *data by codon position (2P and 3P strategies) for both the *ingroup *and *outgroup *datasets. This type of misbehavior seems to be dependent on the characteristics of particular datasets, so that slight changes in taxon sampling can provide correct estimates [64]. Taking advantage from the availability of *nd5 *sequences of *Carabus *in GenBank, we have conducted NP, 2P and 3P partitioning calibration analyses for the expanded *nd5 *dataset (58 sequences; Additional file 1: Table S2). These analyses produced moderate increments in the estimated evolutionary rate for *nd5 *as more partitions were considered, in agreement with the results from other mtDNA genes, supporting the reliability of these estimates.

### Clock calibration with non-coding genes

The effect of methodological choices is markedly higher for non-coding gene fragments than it is for protein coding genes, on both mean rate and node age estimations, but also on their corresponding 95% HPD intervals. Indeed, calibration exercises based on non-coding genes frequently face alignment ambiguity and departures from the molecular clock, which can both jeopardize reliable branch length estimation. These problems increase with evolutionary distance between taxa, thus raising concerns about deep node calibration using slowly evolving ribosomal genes. Several alignment strategies have been proposed to deal with length variation in homologous DNA sequences (see an evaluation of methods in [65]), and discarding ambiguous DNA positions has been also proposed as a complementary method [35]. We examined the effects of the latter procedure on global rate and node age estimation. Expectedly, character removal, especially when applying restrictive culling options (*nogaps *in Gblocks), has a marked effect which is negatively correlated with marker conservation. Highly variable gene fragments, with gappy alignments, can lose a significant amount of phylogenetic information when filtered out of ambiguous alignment positions, and consequently contribute with unreliable calibrations.

As with protein coding genes, there is a strong effect of clock model selection for nuclear ribosomal gene fragments as well. For these markers, SC was unfavored in most cases and its implementation resulted in lower mean values for inferred evolutionary rates and higher ages (up to fourfold) on the estimated TMRCA of *Carabus*, compared to the use of a favored ULN clock. While this behavior is not exclusive of non-coding genes, its prevalence for non-coding data cautions about their use for node age estimation without appropriate accounting for rate heterogeneity in calibration analyses.

## Conclusions

Our study centred on the genus *Carabus *illustrates some of the common problems but also alternative solutions and suitable strategies to molecular clock calibration analyses, exploring the possibility of synergic effects of wrong methodological decision. Mitochondrial genes generally fit the strict clock model of evolution, providing an adequate and simple frame to conduct calibration analyses. However, the combination of several genes including nuclear and mitochondrial fragments results in the best strategy to minimise the effect of both the idiosyncratic behavior of individual markers and analytical aspects on the estimation of evolutionary rates and node ages. But even for mitochondrial genes or combined datasets, rate differences between markers, together with the effect of specific analytical decisions on their estimation, advise against extrapolating rates between different studies. As a summary of the findings reported here, it should be always desirable to check for the appropriate data partition scheme, and for the underlying evolutionary model for each partition; it is also necessary to investigate whether the data really fit a molecular clock previous to any calibration study, applying suitable corrections if needed. The analysis of time on trees should avoid as much as possible regions with dubious homology due to alignment ambiguity, or keep them to a minimum, since gap exclusion has a dramatic effect on branch length estimation and concomitant rates and node ages. Finally, the analyses benefit from enquiry circumscribed to ingroup taxa (and using relatively recent calibration nodes), minimizing the biases introduced by highly divergent lineages and saturation.

## Abbreviations

BF: Bayes factors; LnBF: Natural logarithm of the Bayes factors; HPD: Highest posterior density; SD: Standard deviation; TMRCA: Time to the most recent common ancestor; NP: Non-partitioned data; 2P: Two codon partitions: with 1st and 2nd position together; 3P: Three codon partitions; G-NP: Partitioning by gene with no codon partition; G-2P: Partitioning by gene: with two codon partition of coding genes with 1st and 2nd position together; G-3P: Partitioning by gene: with three codon partition of coding genes; SC: Strict clock; ULN: Uncorrelated log normal clock; Nogaps: Dataset excluding ambiguous character with the *Nogaps *option and default parameters in Gblocks; Allgaps: Idem: applying *Allgaps *option; MIT: Concatenation of mitochondrial genes; NUC: Concatenation of nuclear genes; MIT-NUC: Concatenation of all genes

## Competing interests

The enclosed work has not been under consideration for publication in another journal or book and has been approved by all authors and institutions. All persons entitled to authorship have been so named and all authors have seen and agreed to the submitted version of the manuscript, and declare no competing interests affecting them in the paper under consideration.

## Authors' contributions

CA and JS collected samples. CA and JG-Z conceived the study and designed the analyses, CA gathered the molecular data and performed the analyses. JG-Z, CA and JS wrote the manuscript and all authors read and approved the final version.

## Supplementary Material

Additional file 1 **Additional file**. One PDF file including: A) Supporting Figures and Legends. **Figure S1 to S12**. Phylogenetic trees of the genus *Carabus *obtained with MrBayes, BEAST (*outgroup *dataset) and BEAST (*ingroup *dataset) under the selected parameters. Bars represent 95% confident intervals for the node ages in Ma. Numbers inside nodes represent posterior probabilities. S1*. cox1-A*; S2. *cox1-B*; S3. *cob*; S4. *nd5*; S5*. rrnL*; S6. *LSU-A*; S7. *LSU-B*; S8. *ITS2*; S9. *HUWE1*. S10. MIT; S11. NUC; S12. MIT-NUC. B) Supporting Tables. **Table S1 **Primers used in the molecular clock calibration study of the genus *Carabus*. **Table S2 **Data about *nd5 *sequences and specimens of the genus *Carabus *and related taxa employed to conduct initial calibration analyses. **Table S3 **Calculations for the objective selection of alignments. **Table S4**. Marginal likelihood values in BEAST analyses for individual and combined gene fragments as estimated in Tracer v1.5. **Table S5 **Mean rates of molecular evolution and 95% HPD intervals in calibration analyses on the genus *Carabus*. **Table S6 **Mean ages and 95% HPD intervals in calibration analyses on the genus *Carabus*. C) Supplementary Text D) Supplementary References. Click here for file
