# Supplementary material for: Winding up the molecular clock in the genus Carabus (Coleoptera: Carabidae): assessment of methodological decisions on rate and node age estimation
Source: BMC Evol Biol. 2012 Mar 28;12:40. doi: 10.1186/1471-2148-12-40 (PMC3368785; doi:10.1186/1471-2148-12-40)
Supplement: Additional file 1 — Additional file. One PDF file including: A) Supporting Figures and Legends. Figure S1 to S12. Phylogenetic trees of the genus Carabus obtained with MrBayes, BEAST (outgroup dataset) and BEAST (ingroup dataset) under the selected parameters. Bars represent 95% confident intervals for the node ages in Ma. Numbers inside nodes represent posterior probabilities. S1. cox1-A; S2. cox1-B; S3. cob; S4. nd5; S5. rrnL; S6. LSU-A; S7. LSU-B; S8. ITS2; S9. HUWE1. S10. MIT; S11. NUC; S12. MIT-NUC. B) Supporting Tables. Table S1 Primers used in the molecular clock calibration study of the genus Carabus. Table S2 Data about nd5 sequences and specimens of the genus Carabus and related taxa employed to conduct initial calibration analyses. Table S3 Calculations for the objective selection of alignments. Table S4. Marginal likelihood values in BEAST analyses for individual and combined gene fragments as estimated in Tracer v1.5. Table S5 Mean rates of molecular evolution and 95% HPD intervals in calibration analyses on the genus Carabus. Table S6 Mean ages and 95% HPD intervals in calibration analyses on the genus Carabus. C) Supplementary Text D) Supplementary References. [file 1471-2148-12-40-S1.PDF]

Winding up the molecular clock in the genus Carabus (Coleoptera: Carabidae): assessment of methodological decisions on rate and node age estimates

Supporting Online Material

Carmelo Andújar, José Serrano, Jesús Gómez-Zurita

This PDF file includes:
Figures S1 to S12
Tables S1 to S6
Supplementary text S1
References

Abbreviations: ***BF*** **-** Bayes factor; ***LnBF*** **-** natural logarithm of the Bayes factor; ***HPD*** **-** highest posterior density; ***SD*** **-** standard deviation; ***TMRCA*** **-**Time to the most recent common ancestor.***NP*** **-** no partition; ***2P*** **-** two codon partitions, with 1st and 2nd position together; ***3P*** **-** three codon partitions; ***G-NP*** **-** partitioning by gene with no codon partition; ***G-2P*** **-** partitioning by gene, with two codon partition of coding genes with 1st and 2nd position together; ***G 3P*** **-** partitioning by gene, with three codon partition of coding genes; ***SC*** **-** strict clock; ***ULN*** **-** uncorrelated log normal clock; ***Nogaps*** **-** dataset excluding ambiguous character with the *Nogaps* option and default parameters in Gblocks; ***Allgaps*** **-** idem, applying *Allgaps* option; ***MIT*** **-** concatenation of mitochondrial genes; ***NUC*** **-** concatenation of nuclear genes; ***MIT-NUC*** **-** concatenation of all genes; ***Outgroup dataset*** **-** datasets including out-groups to *Carabus*; ***Ingroup dataset*** **-** without out-groups to *Carabus; **Only coding**:* Excluding the 3' non-coding fragment on the *HUWE1* gene*; **Complete***. No character exclusion.

Table of Contents

A) Supporting Figures and Legends

Figure S1 to S12. Phylogenetic trees of the genus *Carabus* obtained with MrBayes, BEAST (*outgroup* dataset) and BEAST (*ingroup* dataset) under the selected parameters. Bars represent 95% confident intervals for the node ages in Ma. Numbers inside nodes represent posterior probabilities. S1. *cox1-A*; S2. *cox1-B*; S3. *cob*; S4. *nd5*; S5. *rrnL*; S6. *LSU-A*; S7. *LSU-B*; S8. *ITS2*; S9. *HUWE1*. S10. MIT; S11. NUC; S12. MIT-NUC.

B) Supporting Tables

Table S1. Primers used in the molecular clock calibration study of the genus *Carabus*.

Table S2. Data about *nd5* sequences and specimens of the genus *Carabus* and related taxa employed to conduct initial calibration analyses.

Table S3. Calculations for the objective selection of alignments.

Tabla S4. Marginal likelihood values in BEAST analyses for individual and combined gene fragments as estimated in Tracer v1.5.

Table S5. Mean rates of molecular evolution and 95% HPD intervals in calibration analyses on the genus *Carabus*.

Table S6. Mean ages and 95% HPD intervals in calibration analyses on the genus *Carabus*.

C) Supplementary text

D) Supplementary References

A) Supporting Figures and Legends

Figure S1 to S12. Phylogenetic trees of the genus *Carabus* obtained with MrBayes, BEAST (*outgroup* dataset) and BEAST (*ingroup* dataset) under the selected parameters. Bars represent 95% confident intervals for the node ages in Ma. Numbers inside nodes represent posterior probabilities. S1. *cox1-A*; S2. *cox1-B*; S3. *cob*; S4. *nd5*; S5. *rrnL*; S6. *LSU-A*; S7. *LSU-B*; S8. *ITS2*; S9. *HUWE1*. S10. MIT; S11. NUC; S12. MIT-NUC.

Supporting Figure S1

cox1-A

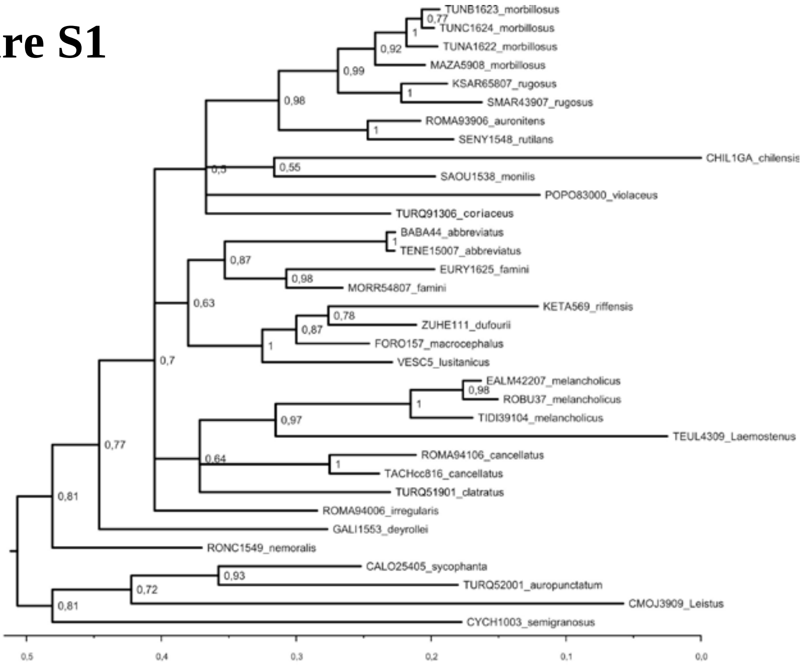

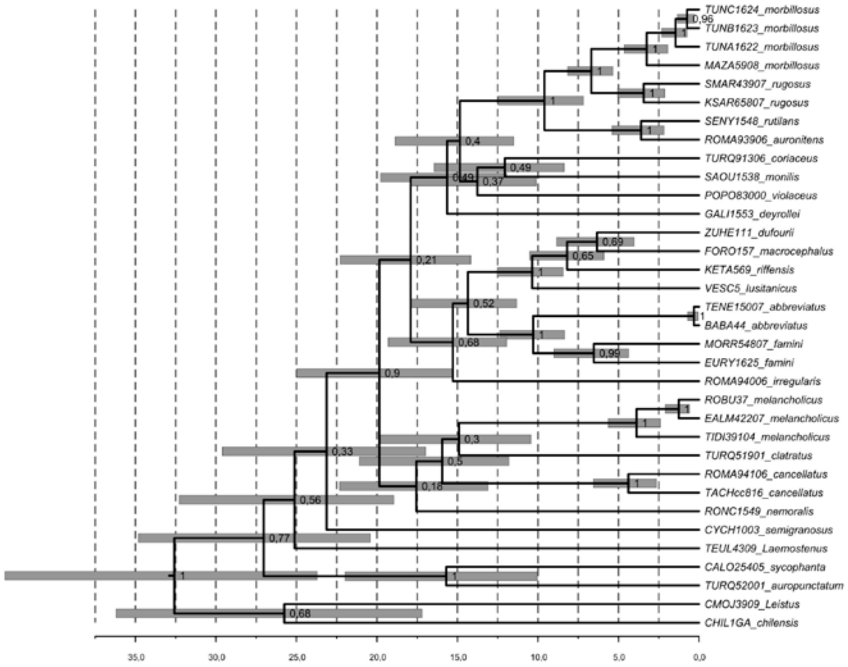

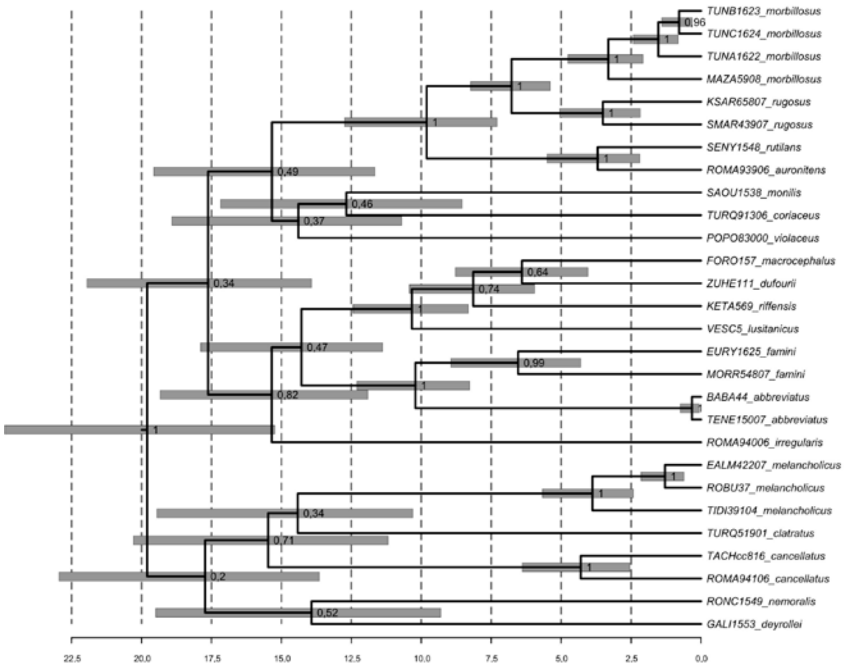

Supporting Figure S2

cox1-B

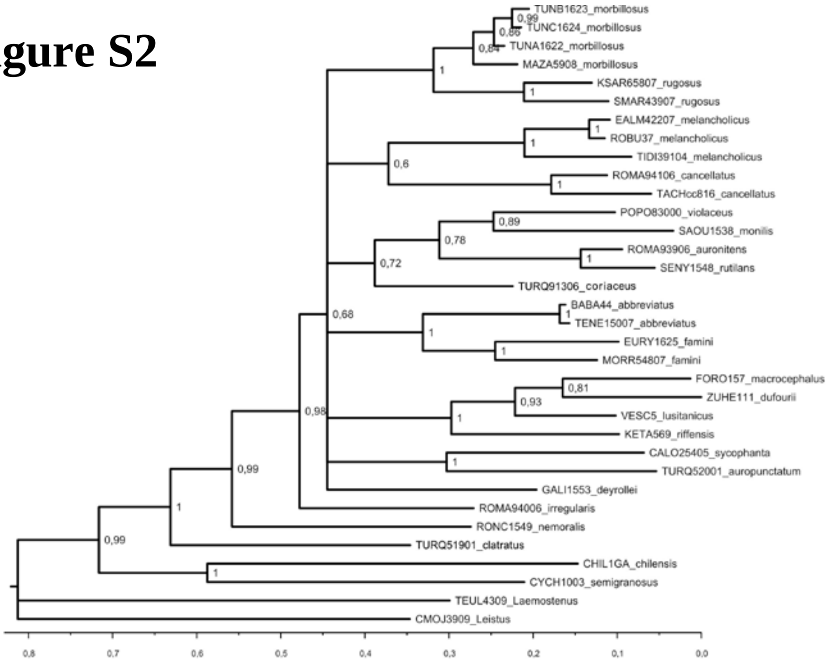

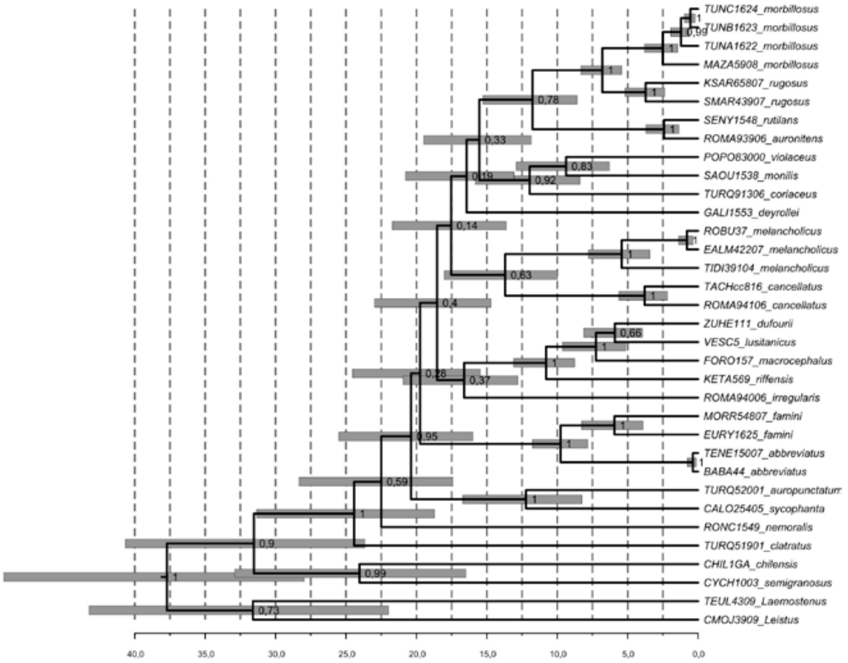

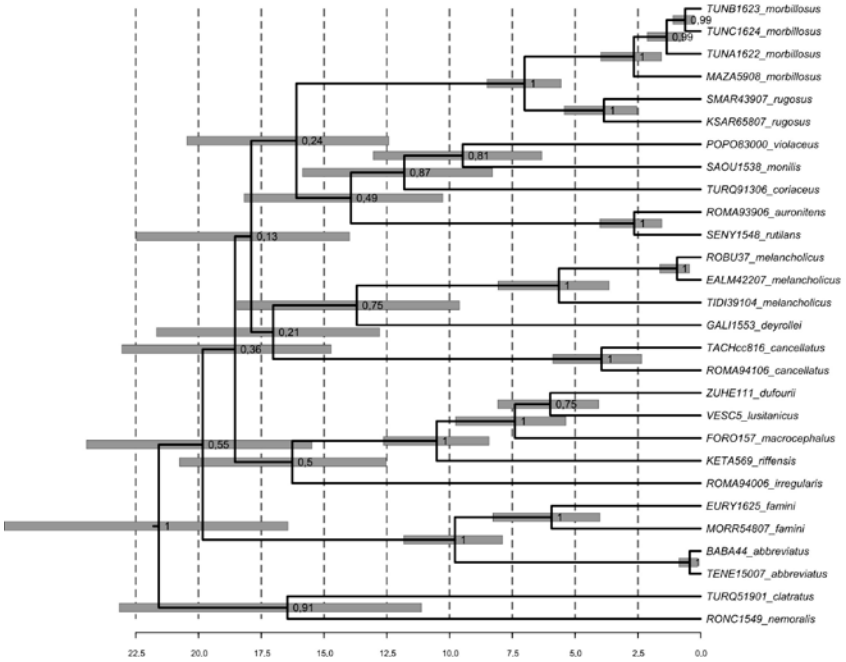

Supporting Figure S3

cob

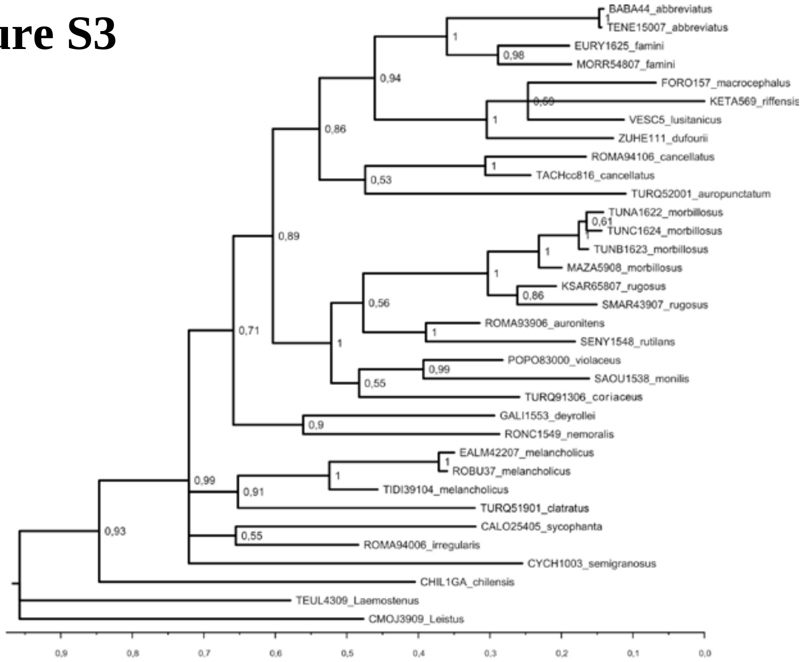

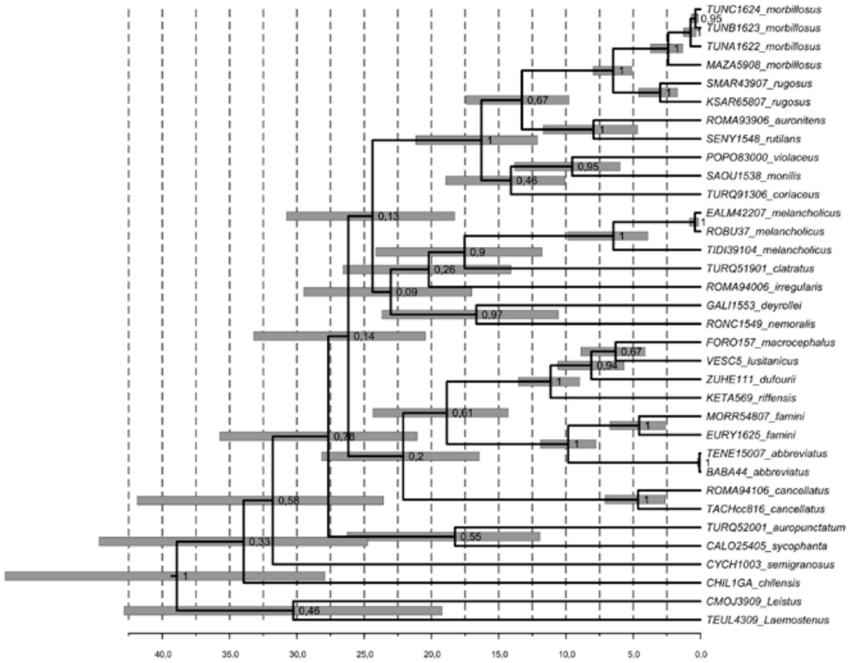

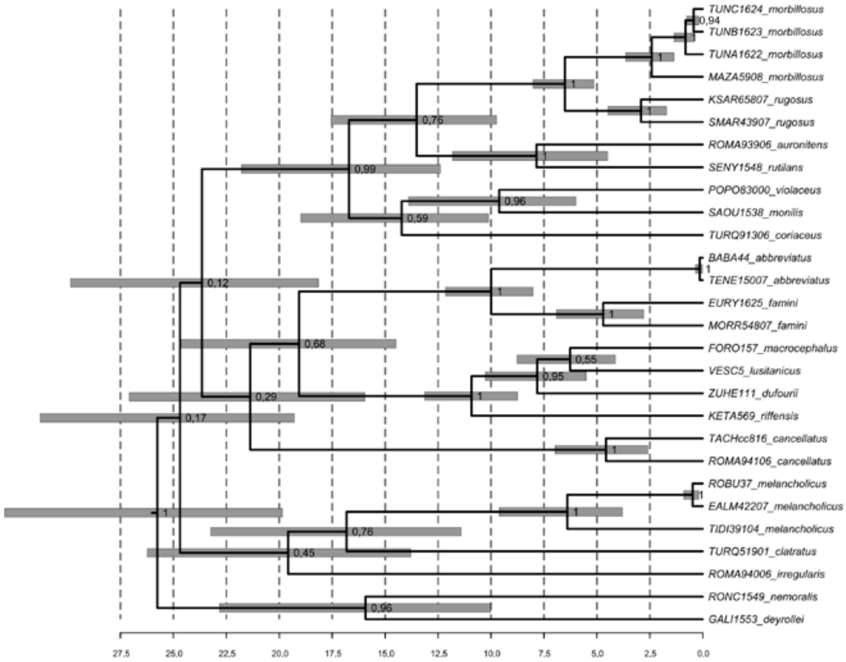

Supporting Figure S4

nd5

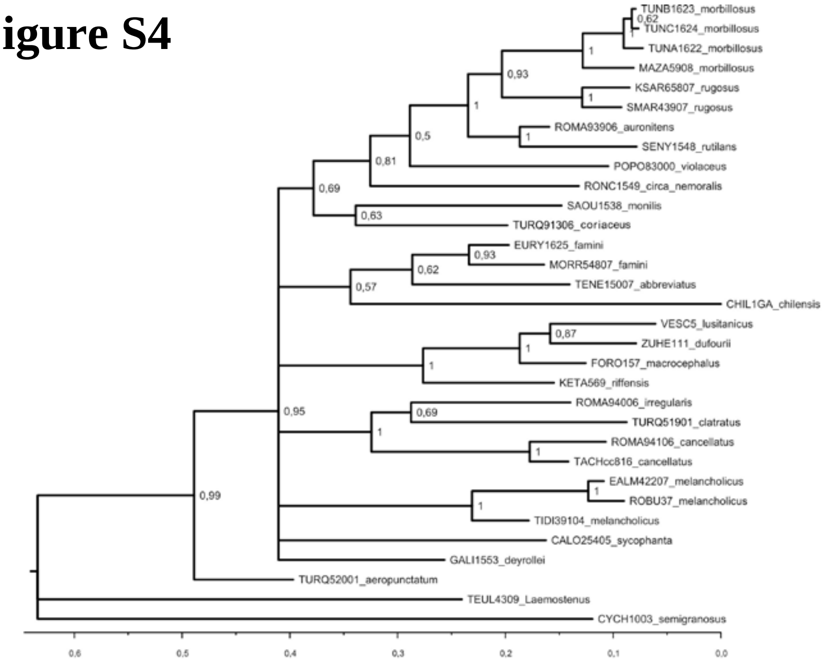

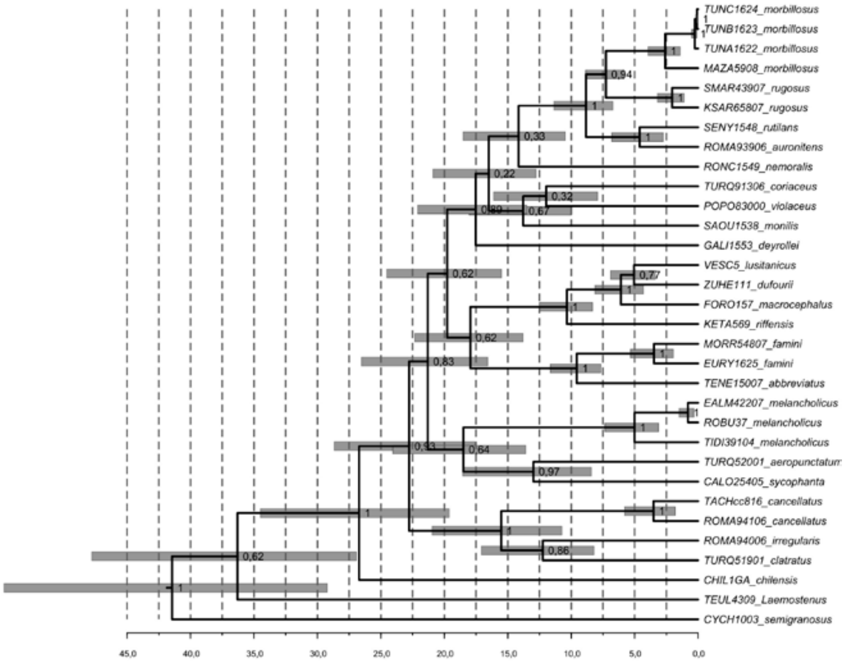

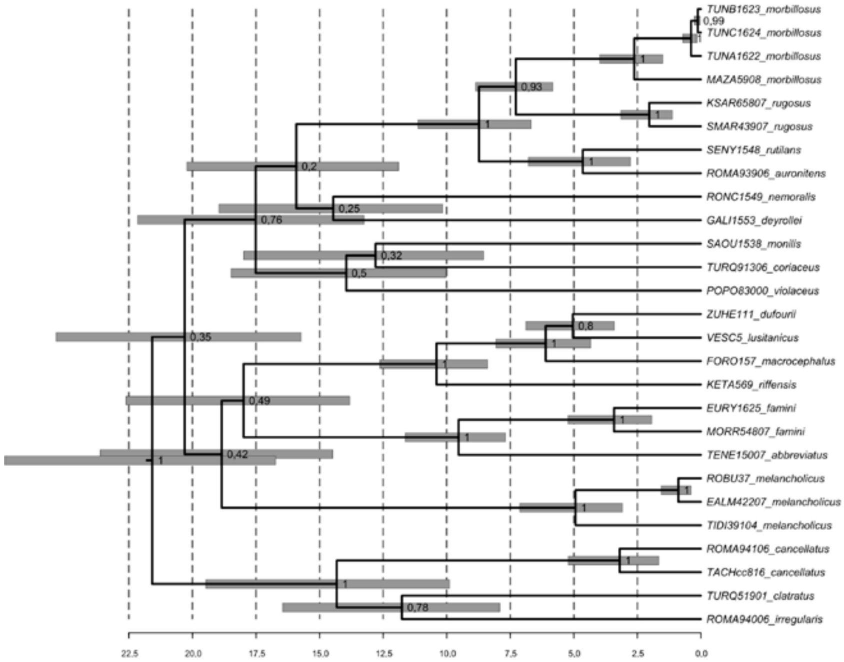

Supporting Figure S5

rrnL

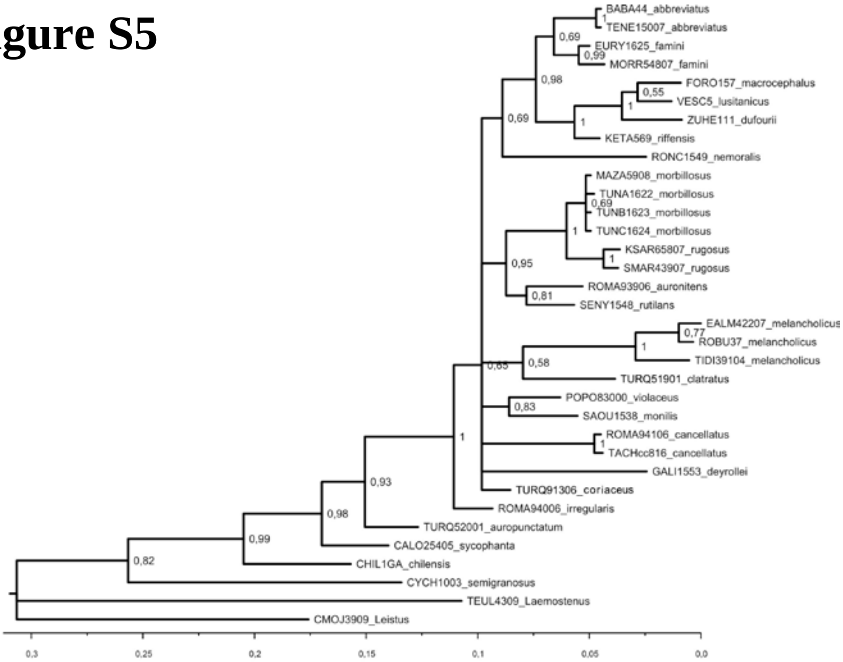

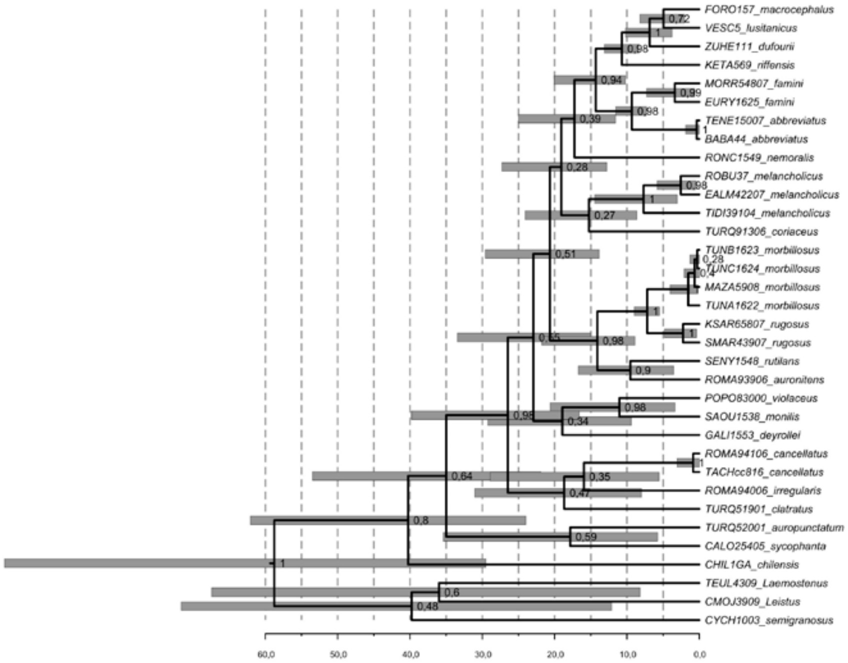

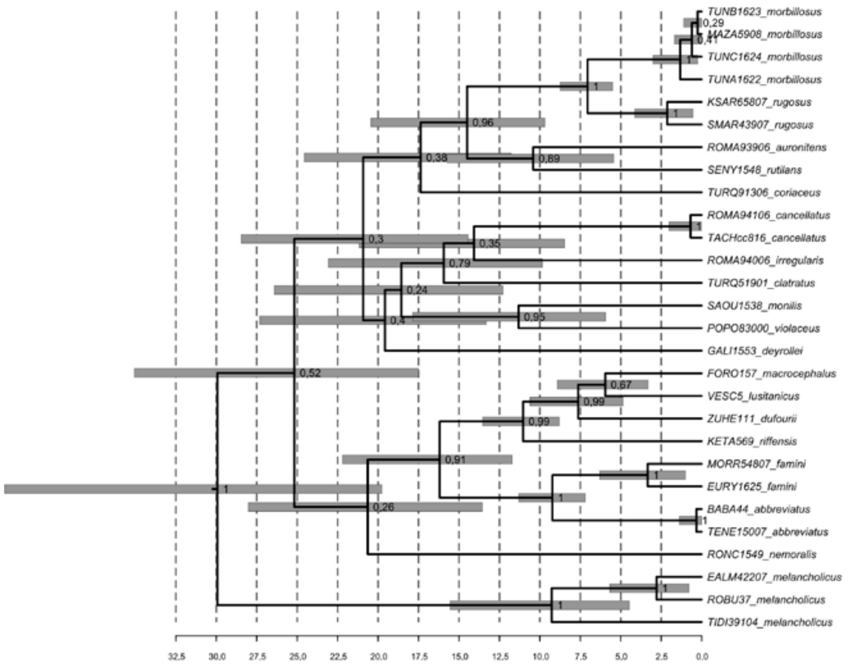

Supporting Figure S6

LSU-A

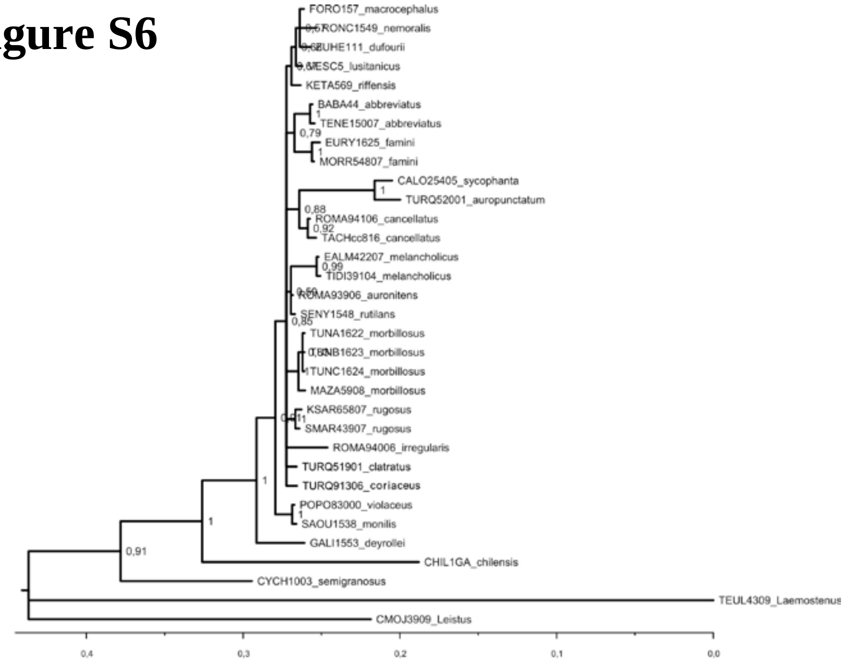

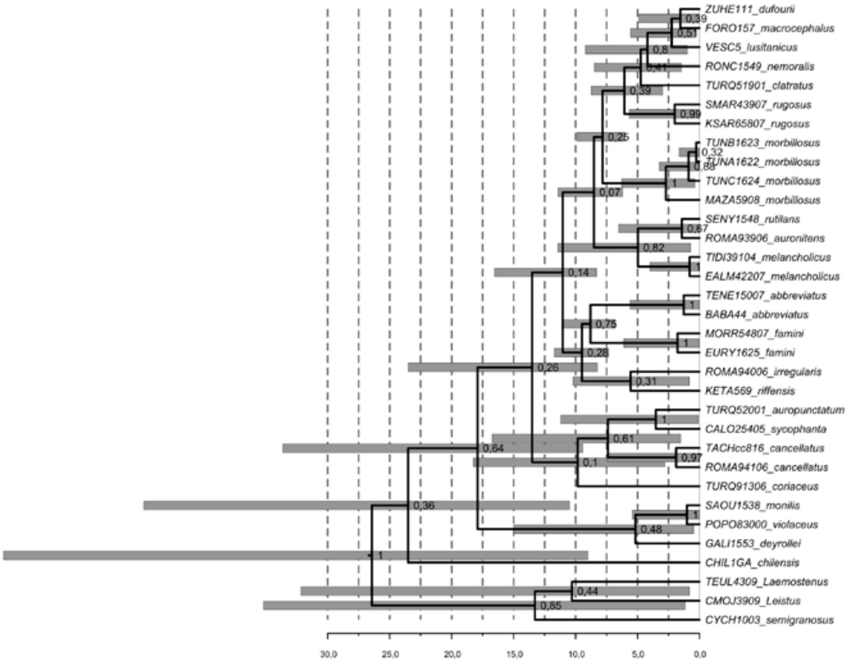

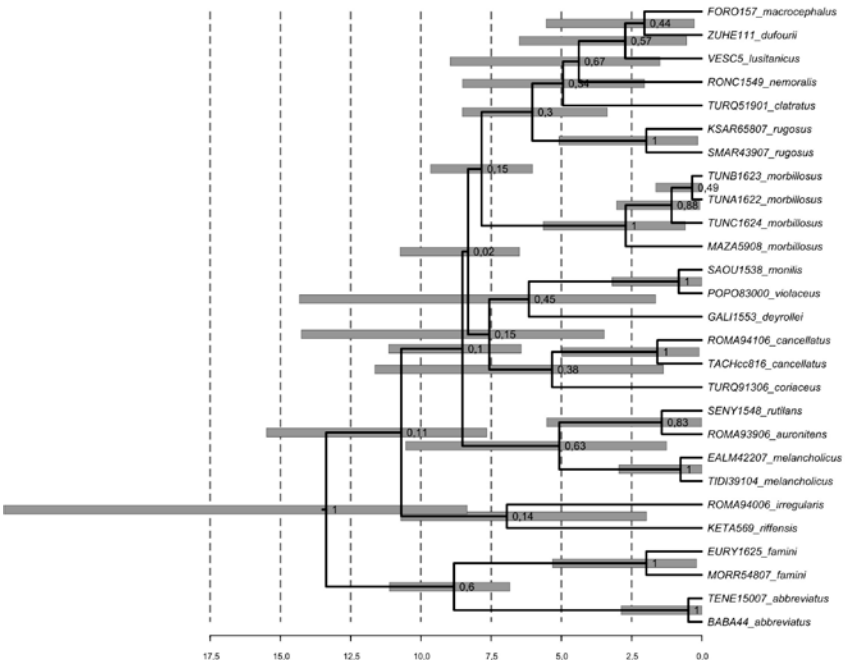

Supporting Figure S7

LSU-B

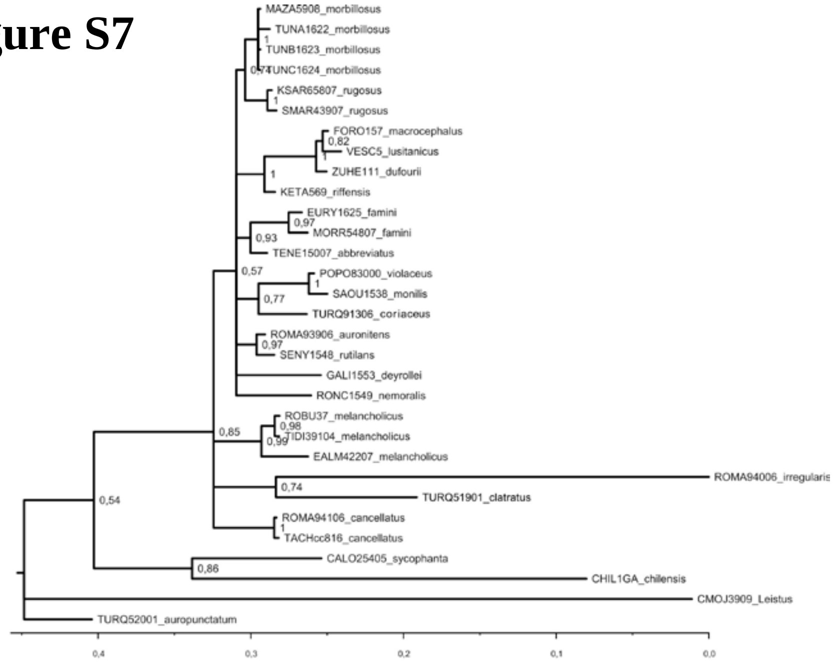

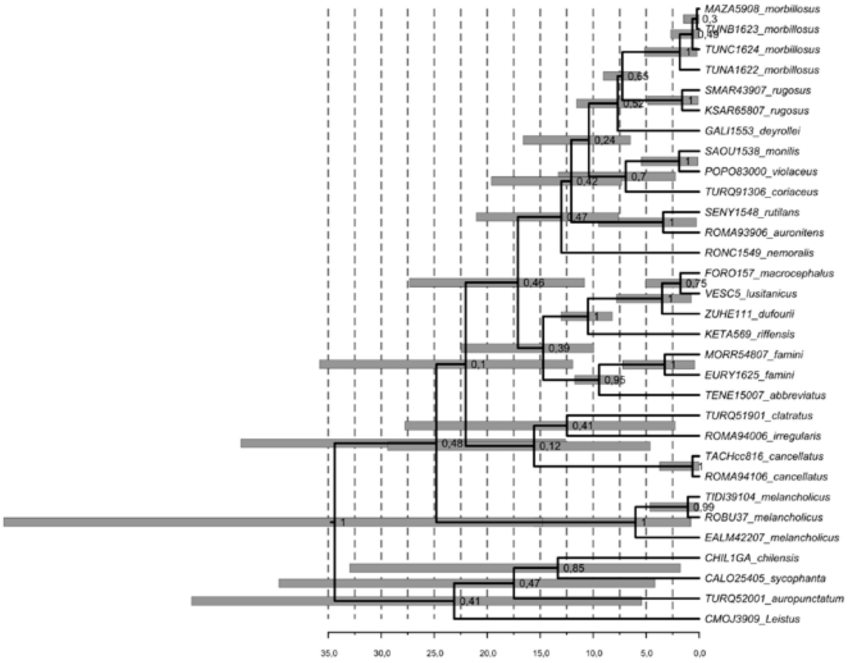

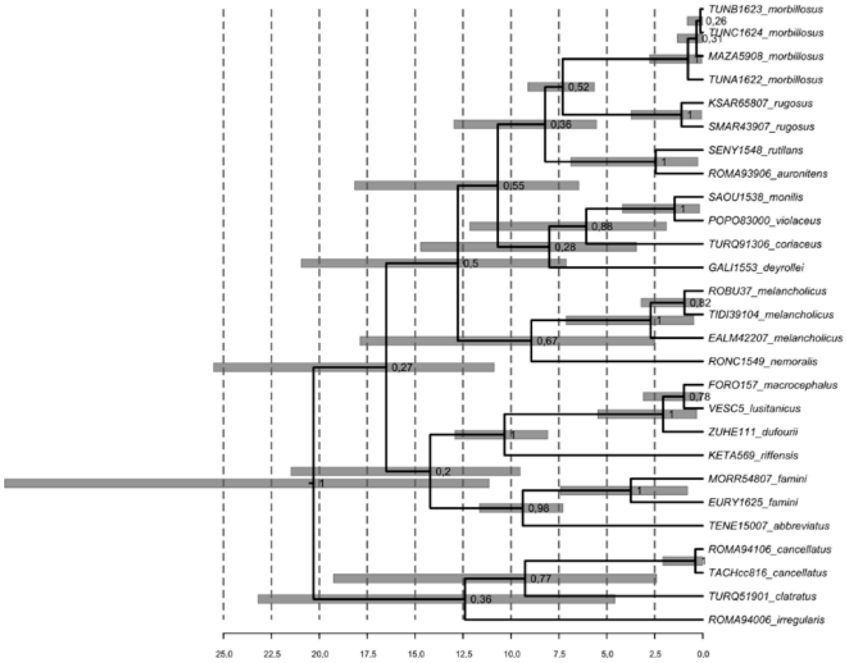

Supporting Figure S8

ITS2

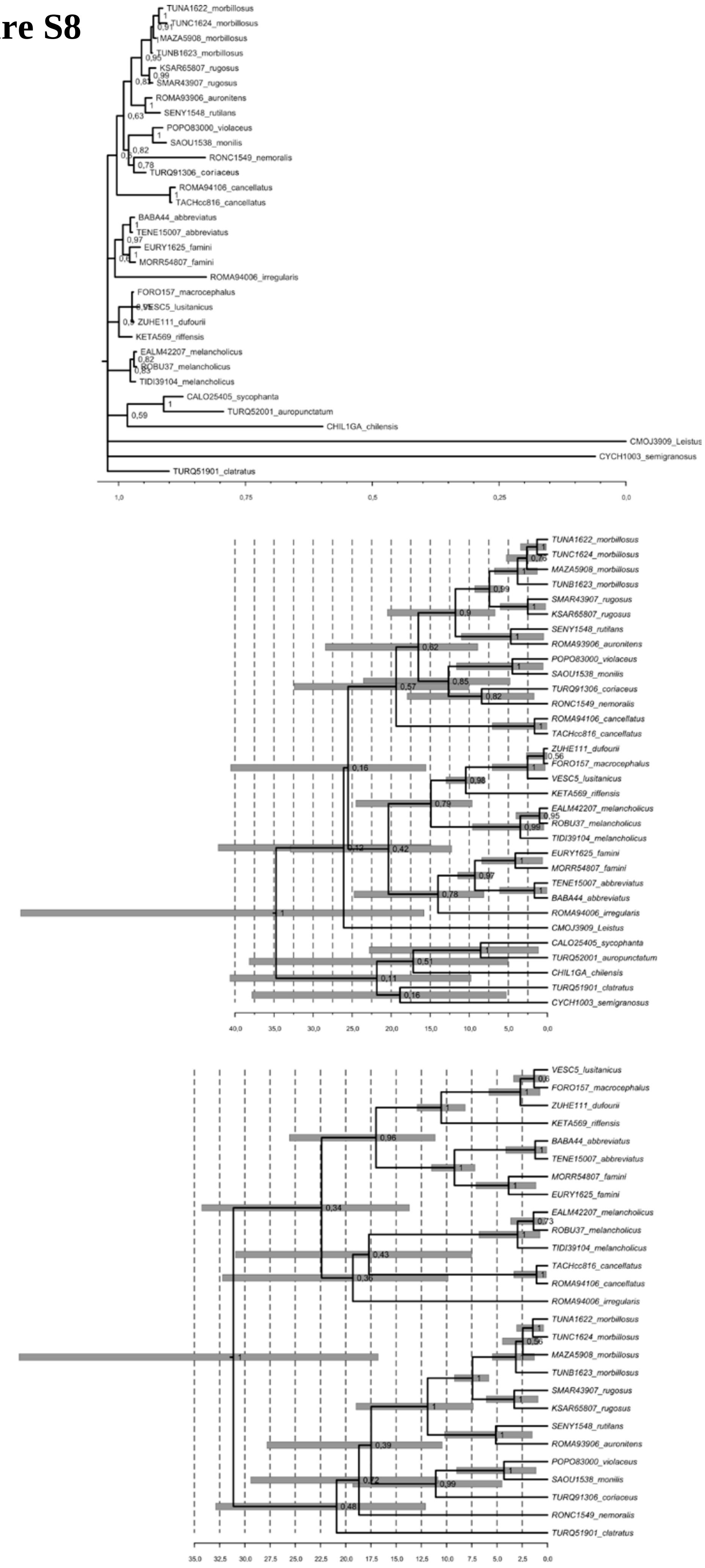

Supporting Figure S9

HUWE1

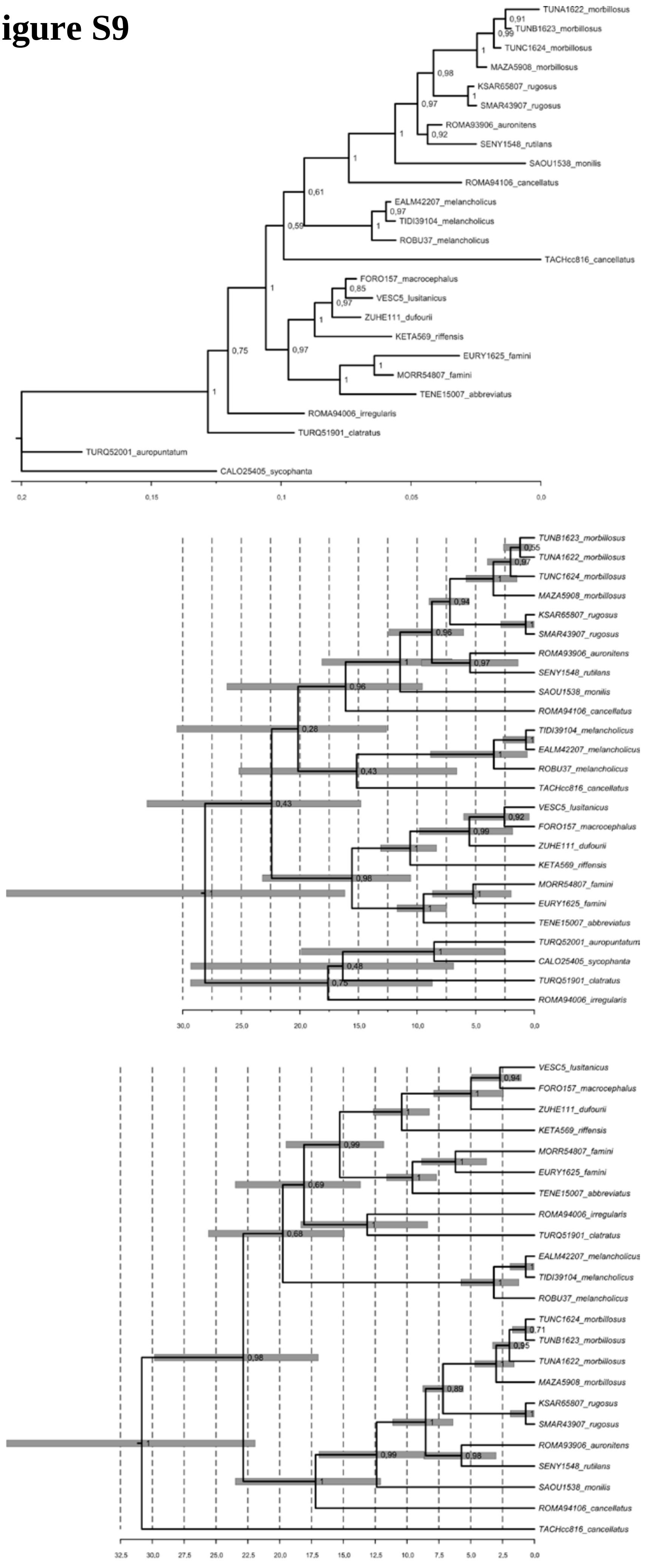

Supporting Figure S10

MIT

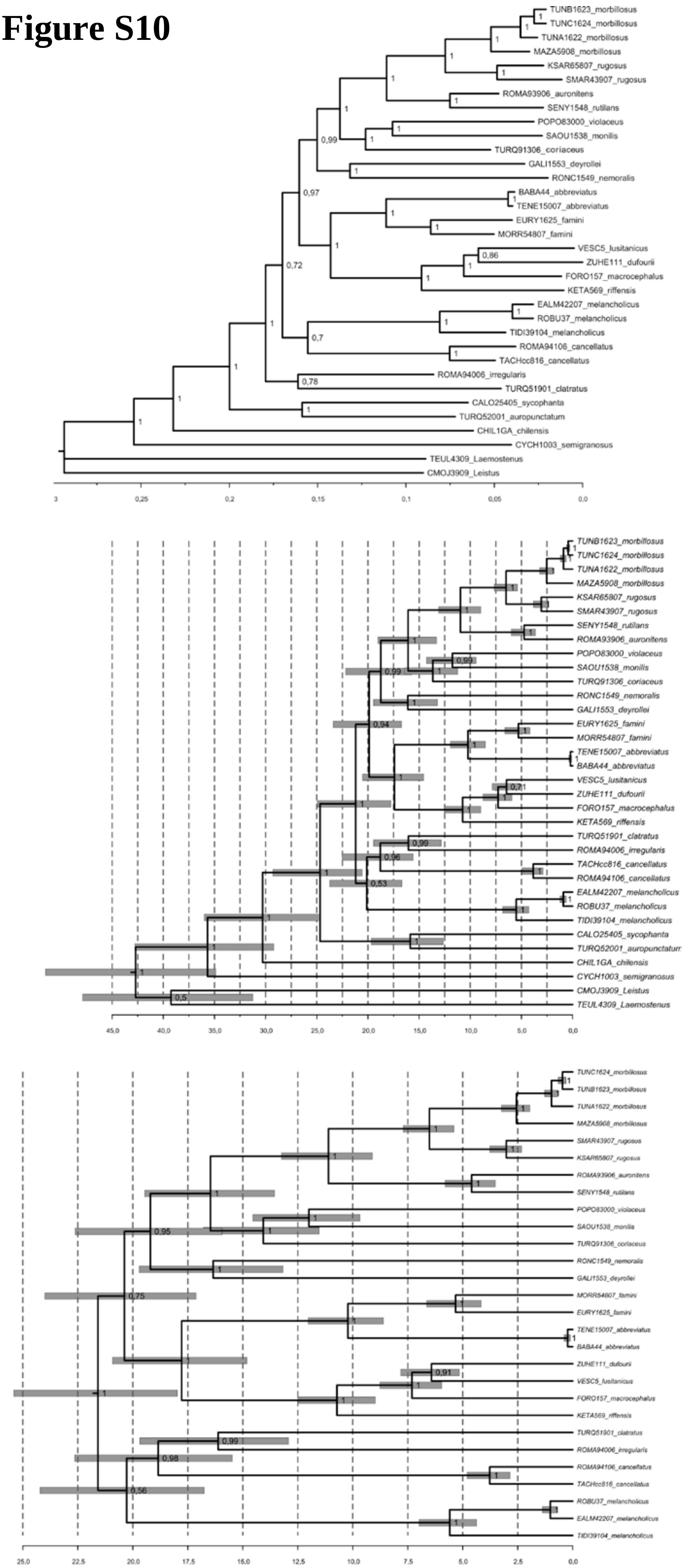

Supporting Figure S11

NUC

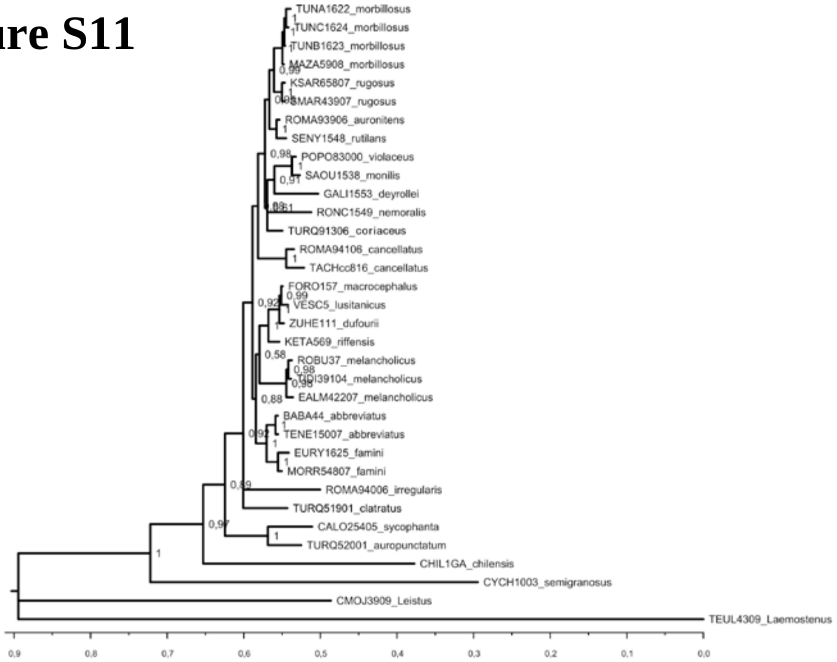

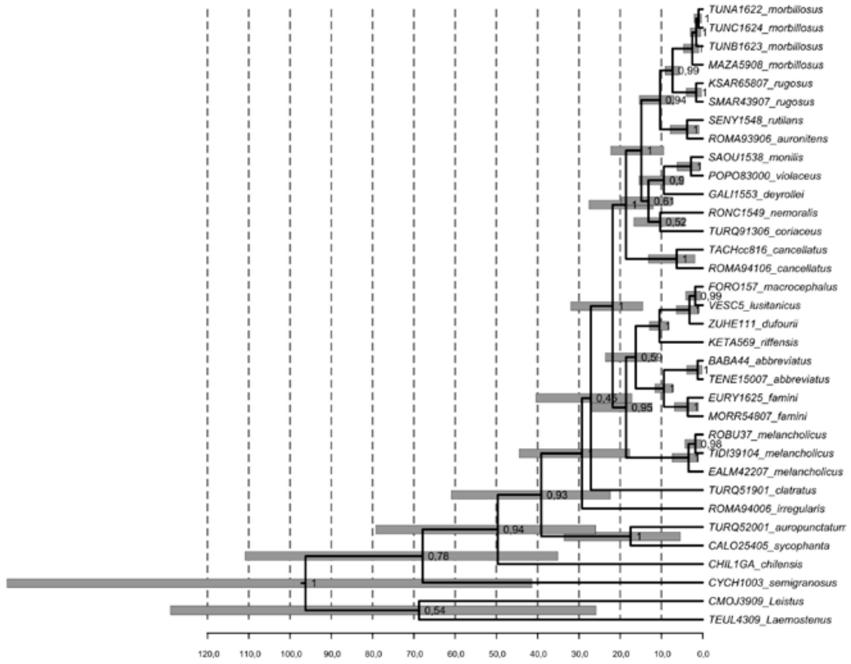

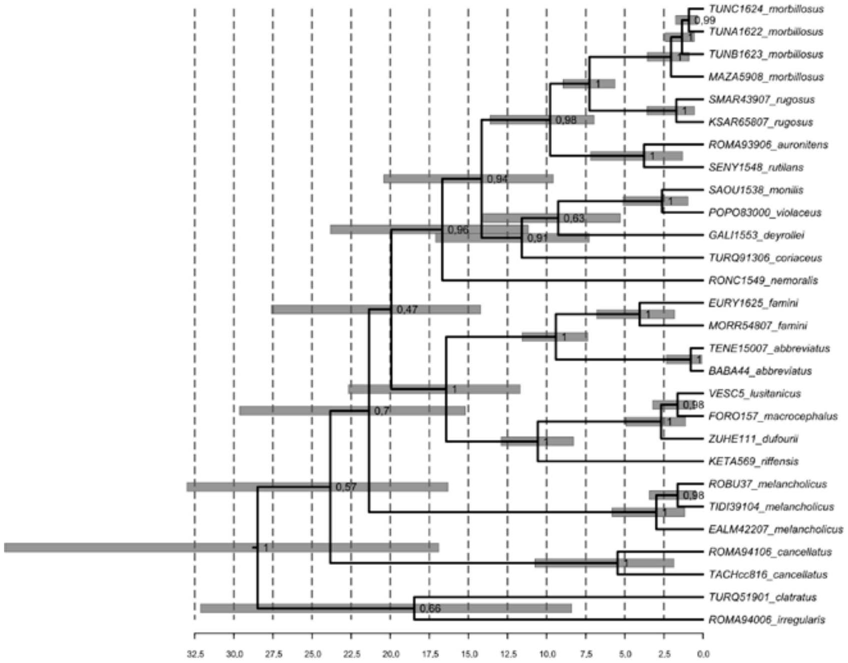

Supporting Figure S12

MIT-NUC

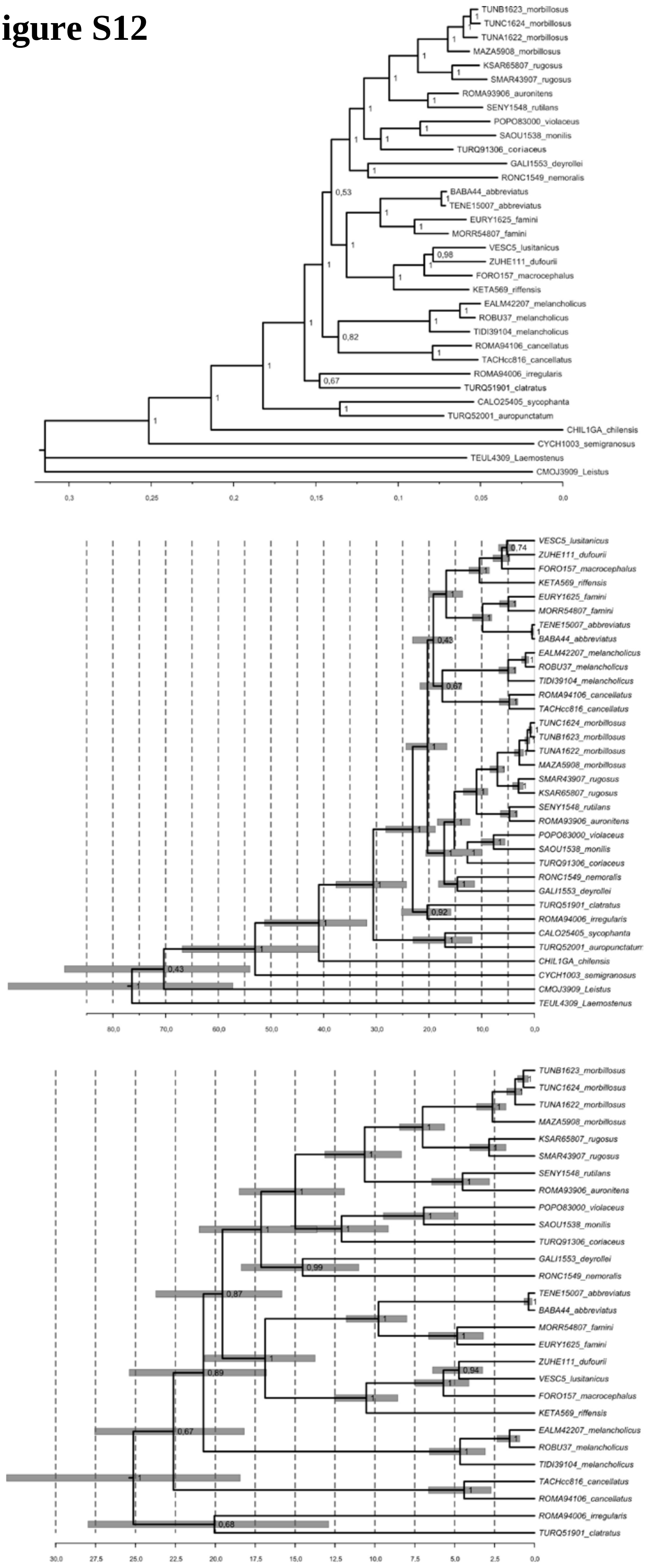

B) Supporting Tables

Table S1. Primers used in the molecular clock calibration study of the genus *Carabus*. F, forward; R, reverse.

Table S2. Data about nd5 sequences and specimens of the genus *Carabus* and related taxa employed to conduct initial calibration analyses.

Table S3. Calculations for the objective selection of alignments of DNA fragments that showed variation in the length of sequences. The alignment that showed the lowest score of incongruence with respect to the unambiguously aligned regions, as measured with R-ILD index, was selected (marked with *). a) *Outgroup* dataset. b) *Ingroup* dataset.

Tabla S4. Marginal likelihood values in BEAST analyses for individual and combined gene fragments as estimated in Tracer v1.5. Selected analyses are shown in bold. **a)** Individual gene fragments with the *ingroup* datasets. **b)** Individual gene fragments with the *outgroup* datasets. **c)** Combined matrixes with *ingroup* and *outgroup* datasets.

Table S5. Mean rates of molecular evolution and 95% HPD intervals in calibration analyses on the genus *Carabus* for: a) Protein coding genes. b) Non coding genes. c) Combined datasets. O.G. *Outgroup* dataset; I.G. *Ingroup* dataset

Table S6. Mean ages and 95% HPD interval in calibration analyses on the genus *Carabus* for: a) Protein coding gene. b) Non coding genes. c) Combined datasets. O.G. *Outgroup* dataset; I.G. *Ingroup* dataset

Table S1. Primers used in the molecular clock calibration study of the genus *Carabus*. F, forward; R, reverse.

Type DNA	Gene/ fragment	Length	Primer	S	Primer sequence (5'- 3')	Described in:
Mitochondrial protein coding	*nd5*	903	V1.06-1	F	CCTGTTTCTGCTTTAGTTCA	(Su *et al.*, 1996b)
			V1.04-4	R	GTCATACTCTAAATATAAGCTA	(Su *et al.*, 1996b)
Mitochondrial protein coding	*cox1*-A	576	lco1490	F	GGTCAACAAATCATAAAGATATTGG	(Folmer *et al.*, 1994)
			hco2198	R	TAAACTTCAGGGTGACCAAAAAATCA	(Folmer *et al.*, 1994)
Mitochondrial protein coding	*cox1*-B	768	Jerry (M202)	F	CAACATTTATTTTGATTTTTTGG	(Simon *et al.*, 1994)
			Pat (M70)	R	TCCA(A)TGCACTAATCTGCCATATTA	(Simon *et al.*, 1994)
Mitochondrial protein coding	*cob*	666	CP1	F	GATGATGAAATTTTGGATC	(Harry *et al.*, 1998)
			CB2	R	ATTACACCTCCTAATTTATTAGGAAT	(Crozier and Crozier, 1992)
Mitochondrial ribosomal	*rrnl*	718-733	16SaR (M14)	F	CGCCTGTTTAWCAAAAACAT	(Simon *et al.*, 1994)
			16s-ND1a (M223)	R	GGTCCCTTACGAATTTGAATATATCCT	(Simon *et al.*, 1994)
Nuclear ribosomal	*LSU* -A	951-1065	LS58F (D1)	F	GGGAGGAAAAGAAACTAAC	(Ober, 2002)
			LS998R (D3)	R	GCATAGTTCACCATCTTTC	(Ober, 2002)
Nuclear ribosomal	*LSU*-B	760-970	d67F	F	GGAGTGTGTAACAACTCACCTGCCG	-
			28s-rD7b1 (d67R)	R	GACTTCCCTTACCTACAT	(Whiting, 2002)
Nuclear ribosomal	*ITS2*	543-892	5.8sF	F	GTGAATTCTGTGAACTGCAGGACACATGAAC	(Porter and Collins, 1991)
			28sR	R	ATGCTTAAATTTAGGGGGTA	(Porter and Collins, 1991)
Nuclear protein coding	HUWE1 (Anonymous)	542-560	gwnck1	F	GTGACGAACAAGAAGATATGG	-
			Carck2	R	GTGGTTCGCATCTCAACAGA	(Sota & Vogler, 2001)

Table S2. Data about nd5 sequences and specimens of the genus *Carabus* and related taxa employed to conduct initial calibration analyses.

Specie	Locality	Voucher	GB_AN	Source
Abax parallelepipedus	Italy, Laghi, Mt. Lessini		AF190044	(Düring and Brücker, 2000)
Cychrus morawitzi	Japan		D50347	(Su *et al.*, 1996a)
Pamborus opacus	Australia, Queensland		AB109862	(Su *et al.*, 2004)
Pamborus viridis	Australia, Wollomombi		AY722993	(Sota *et al.*, 2005)
Maoripamborus fairburni	New Zealand, Northland		AY722988	(Sota *et al.*, 2005)
Ceroglossus chilensis	Chile, Chiloe		AB057584	(Okamoto *et al.*, 2001)
Calosoma aeropunctatum	Turkey, Kars	TURQ52001	JQ689870	
Carabus (Acoptolabrus) schrencki	Russia, Nakhodka Primorsky		AB041080	(Tominaga *et al.*, 2000)
C. (Archicarabus) nemoralis	Spain, Navarra	RONC1549	JQ689858	
C. (Tachypus) auratus	France, Burgundy		D86204	(Su *et al.*, 1996b)
C. (Tachypus) cancellatus emarginatus	Italy, Piemonte		AB092702	(Su *et al.*, 2003)
C. (Tachypus) cancellatus graniger	Romania, Judet Caras-Severin		AB092700	(Su *et al.*, 2003)
C. (Carabus) deyrollei	Spain, Lugo	GALI1553	JQ689848	
C. (Chrysocarabus) rutilans	Spain, Tarragona	SENY1548	JQ689860	
C. (Ctenocarabus) galicianus	Spain, La Coruna		AB101009	(Kim *et al.*, 2003)
C. (Rhabdotocarabus) melancholicus	Morocco, Jebel el Habib		AF231702	(Arndt *et al.*, 2000)
C. (Rhabdotocarabus) melancholicus	Spain, Pontevedra		AB101007	(Kim *et al.*, 2003)
C. (Rhabdotocarabus) melancholicus	Spain, Cadiz	EALM42207	JQ689845	
C. (Rhabdotocarabus) melancholicus	Morocco, Tidiquin	TIDI39104	JQ689865	
C. (Damaster) blaptoides babaianus	Japan, Fukushima pref.		AB013932	(Su *et al.*, 1998)
C. (Damaster) blaptoides blaptoides	Japan, Shiga pref.		AB013956	(Su *et al.*, 1998)
C. (Damaster) blaptoides oxuroides	Japan, Chiba pref.		AB013945	(Su *et al.*, 1998)
C. (Damaster) blaptoides rugipennis	Japan, Hokkaido		AB013860	(Su *et al.*, 1998)
C. (Eurycarabus) famini favieri	Morocco, Kenitra		AF231689	(Prüser *et al.*, 2000)
C. (Eurycarabus) famini	Morocco, Tanout ou Fillali		AF231690	(Prüser *et al.*, 2000)
C. (Eurycarabus) famini	Morocco, El Alia	EURY1625	JQ689846	
C. (Eurycarabus) famini	Morocco, Rif Mountains	MORR54807	JQ689853	
C. (Eurycarabus) genei	Italy, Sardinia, Macomer		AF231691	(Prüser *et al.*, 2000)
C. (Isiocarabus) miaorum	China, Mt. Miao		AB041053	(Tominaga *et al.*, 2000)
C. (Leptocarabus) arboreus	Japan, Fukushima		AB047415	(Kim *et al.*, 2000a)
C. (Leptocarabus) hiurai	Japan, Saijo		AB031436	(Kim *et al.*, 2000b)
C. (Leptocarabus) koreanus	Korea, Gonjiam		AB031451	(Kim *et al.*, 2000b)
C. (Leptocarabus) kyushuensis	Japan, Kumamoto		AB047474	(Kim *et al.*, 2000a)
C. (Leptocarabus) procerulus	Japan, Iwate		AB047445	(Kim *et al.*, 2000a)
C. (Limnocarabus) clatratus	Turkey, Kars	TURQ51901	JQ689869	
C. (Macrothorax) morbillosus	Italy, Sardinia		AB101013	(Kim *et al.*, 2003)
C. (Macrothorax) morbillosus	Spain, Murcia	MAZA5908	JQ689852	
C. (Macrothorax) morbillosus	Tunisia		AB101012	(Kim *et al.*, 2003)
C. (Macrothorax) morbillosus	Tunisia, Sejenane	TUNA1622	JQ689866	
C. (Macrothorax) morbillosus	Tunisia, Bazia	TUNB1623	JQ689867	
C. (Macrothorax) morbillosus	Tunisia, El Alia	TUNC1624	JQ689868	
C. (Macrothorax) rugosus	Morocco, Asilah	ARIL1550	JQ739173	
C. (Macrothorax) rugosus	Morocco, Ksar-el-kebir	KSAR65807	JQ689851	
C. (Macrothorax) rugosus	Spain, Cadiz	SMAR43907	JQ689861	
C. (Mesocarabus) dufourii	Spain, Cordoba	ZUHE111	JQ689873	
C. (Mesocarabus) lusitanicus	Spain, Ciudad Real	VESC5	JQ689872	
C. (Mesocarabus) macrocephalus	Spain, La Coruna	FORO157	JQ689847	
C. (Mesocarabus) riffensis	Morocco, El Biutz	EBUI1397	JQ739174	
C. (Mesocarabus) riffensis	Morocco, Ketama	KETA569	JQ689850	
C. (Nesaeocarabus) abbreviatus	Spain, Tenerife		AB092707	(Su *et al.*, 2003)
C. (Nesaeocarabus) abbreviatus	Spain, Tenerife		AF231696	(Prüser *et al.*, 2000)
C. (Nesaeocarabus) coarctatus	Spain, Gran Canaria		AF231692	(Prüser *et al.*, 2000)
C. (Ohomopterus) albrechti	Japan, Hokkaido		AF219430	(Sota and Vogler, 2001)
C. (Ohomopterus) arrowianus	Japan, Aichi		AF219463	(Sota and Vogler, 2001)
C. (Ohomopterus) dehaanii	Japan, Osaka		AF219439	(Sota and Vogler, 2001)
C. (Ohomopterus) insulicola	Japan, Nagano		AF219469	(Sota and Vogler, 2001)
C. (Ohomopterus) japonicus	Japan, Shimane		AF219449	(Sota and Vogler, 2001)
C. (Ohomopterus) yaconinus	Japan, Mt. Kagawa		AF219452	(Sota and Vogler, 2001)

Table S3. Calculations for the objective selection of alignments of DNA fragments that showed variation in the length of sequences. The alignment that showed the lowest score of incongruence with respect to the unambiguously aligned regions, as measured with R-ILD index, was selected (marked with *). a) *Outgroup* dataset. b) *Ingroup* dataset.

a)

**With Outgroups**	**FFT-NS-i**			**E-INS-i**			**L-INS-i**			**G-INS-i**			**Q-INS-i**		
	1PAM	20PAM	200PAM	1PAM	20PAM	200PAM	1PAM	20PAM	200PAM	1PAM	20PAM	200PAM	1PAM	20PAM	200PAM
**rrnl**															
Chars	**734**	**734**	**734**	**734**	**734**	**734**	**734**	**734**	**734**	**734**	**734**	**734**	**735**	**735**	**735**
L	465	465	464	464	464	465	464	464	465	470	470	471	470	471	469
Lunamb	6543	6543	6543	6543	6543	6543	6543	6543	6543	6543	6543	6543	6543	6543	6543
Lcomb	7017	7017	7018	7017	7017	7018	7017	7017	7018	7023	7023	7024	7022	7024	7023
ILD_{index}	0.00128*	0.00128*	0.00156	0.00142	0.00142	0.00142	0.00142	0.00142	0.00142	0.00142	0.00142	0.00142	0.00128*	0.00142	0.00156
$R\text{-}ILD_{index}$	0.5625*	0.5625*	0.64705	0.58823	0.58823	0.625	0.58823	0.58823	0.625	0.90909	0.90909	1	0.81818	1	0.91666
**d13**															
Chars	**1113**	**1116**	**1116**	**1125**	**1110**	**1117**	**1119**	**1112**	**1120**	**1116**	**1119**	**1124**	**1096**	**1096**	**1116**
L	941	938	957	937	926	913	916	912	908	934	932	933	904	905	902
Lunamb	6498	6498	6498	6498	6498	6498	6498	6498	6498	6498	6498	6498	6498	6498	6498
Lcomb	7461	7459	7479	7454	7444	7426	7433	7433	7424	7451	7451	7449	7419	7421	7417
ILD_{index}	0.00294	0.00308	0.00320	0.00254	0.00268	0.00201	0.00255	0.00309	0.00242	0.00255	0.00281	0.00241	0.00229*	0.00242	0.00229*
$R\text{-}ILD_{index}$	0.55	0.53488	1	0.43181	0.36363	0.22058	0.29230	0.33333	0.24657	0.40425	0.42857	0.375	0.22077	0.23684	0.21518*
**d67**															
Chars	**1052**	**1063**	**1052**	**1056**	**1065**	**1075**	**1060**	**1073**	**1068**	**1051**	**1073**	**1068**	**1117**	**1146**	**1133**
L	1970	1922	1976	1950	2039	1953	1844	1845	1836	1879	1864	1842	1771	1776	1748
Lunamb	5715	5715	5715	5715	5715	5715	5715	5715	5715	5715	5715	5715	5715	5715	5715
Lcomb	7725	7672	7719	7736	7825	7701	7602	7600	7584	7629	7621	7590	7521	7526	7491
ILD_{index}	0.00517	0.00456	0.0036*	0.00917	0.00907	0.00428	0.00565	0.0052	0.00435	0.00458	0.00551	0.00434	0.00465	0.00465	0.00373
$R\text{-}ILD_{index}$	0.28571	0.18617	0.20895	0.44375	1	0.21019	0.16165	0.1509	0.12043	0.15151	0.17073	0.12313	0.10324	0.10479	0.07734*
**ITS2**															
Chars	**1212**	**1199**	**1277**	**1284**	**1327**	**1225**	**1271**	**1246**	**1177**	**1283**	**1310**	**1184**	**1264**	**1242**	**1273**
L	2785	2687	2808	2871	2878	2812	2789	2849	2772	2792	2795	2770	2718	2705	2719
Lunamb	5896	5896	5896	5896	5896	5896	5896	5896	5896	5896	5896	5896	5896	5896	5896
Lcomb	8712	8623	8732	8793	8802	8756	8727	8795	8702	8721	8725	8690	8631	8623	8638
ILD_{index}	0.00355	0.00463	0.0032	0.00295	0.00318	0.00548	0.00481	0.0056	0.00390	0.00378	0.00389	0.00276	0.00196*	0.00255	0.00266
$R\text{-}ILD_{index}$	0.25619	0.18264	0.28571	0.74285	1	0.51063	0.35897	0.8771	0.25373	0.28947	0.30630	0.17647	0.09042*	0.10945	0.12299
**HUWE1**															
Chars	**677**	**677**	**676**	**679**	**682**	**680**	**679**	**682**	**680**	**679**	**682**	**680**	**681**	**681**	**682**
L	564	512	562	526	512	527	526	512	527	526	512	525	512	512	513
Lunamb	4097	4097	4097	4097	4097	4097	4097	4097	4097	4097	4097	4097	4097	4097	4097
Lcomb	4678	4625	4672	4640	4626	4639	4640	4626	4639	4640	4626	4637	4621	4621	4622
ILD_{index}	0.00363	0.00345	0.00278	0.00366	0.00367	0.00323	0.00366	0.00367	0.00323	0.00366	0.00367	0.00323	0.00259*	0.00259*	0.00259*
$R\text{-}ILD_{index}$	1	0.23188	0.68421	0.30909	0.24637	0.27777	0.30909	0.24637	0.27777	0.30909	0.24637	0.26785	0.17391*	0.17391*	0.17647

b)

INGROUP	FFT-NS-i			E-INS-i			L-INS-i			G-INS-i			Q-INS-i		
	1PAM	20PAM	200PAM	1PAM	20PAM	200PAM	1PAM	20PAM	200PAM	1PAM	20PAM	200PAM	1PAM	20PAM	200PAM
**rrnl**															
Chars	**733**	**733**	**733**	**733**	**733**	**733**	**733**	**733**	**733**	**733**	**733**	**733**	**733**	**733**	**733**
L	275	275	275	274	274	274	274	274	274	280	280	280	283	283	280
Lunamb	4622	4622	4622	4622	4622	4622	4622	4622	4622	4622	4622	4622	4622	4622	4622
Lcomb	4905	4905	4906	4905	4905	4905	4905	4905	4905	4911	4911	4911	4913	4913	4911
ILD$_{index}$	0.00163	0.00163	0.00183	0.00183	0.00183	0.00183	0.00183	0.00183	0.00183	0.00183	0.00183	0.00183	0.00162*	0.00162*	0.00183
R-ILD$_{index}$	0.5*	0.5*	0.5625	0.52941	0.52941	0.52941	0.52941	0.52941	0.52941	0.81818	0.81818	0.81818	1	1	0.81818
**d13**															
Chars	**965**	**965**	**965**	**968**	**968**	**970**	**968**	**968**	**970**	**968**	**970**	**970**	**966**	**966**	**969**
L	201	201	201	197	197	197	197	197	197	220	222	220	207	207	211
Lunamb	4575	4575	4575	4575	4575	4575	4575	4575	4575	4575	4575	4575	4575	4575	4575
Lcomb	4785	4785	4785	4786	4786	4782	4786	4786	4782	4809	4810	4805	4793	4793	4797
ILD$_{index}$	0.00188*	0.00188*	0.00188*	0.00292	0.00292	0.00209	0.00292	0.00292	0.00209	0.00291	0.00270	0.00208	0.00229	0.00229	0.00229
R-ILD$_{index}$	0.26470	0.26470	0.26470	0.36842	0.36842	0.26315*	0.36842	0.36842	0.26315	0.93333	1	0.66666	0.39285	0.39285	0.45833
**d67**															
Chars	**1079**	**1066**	**1085**	**1071**	**1072**	**1059**	**1049**	**1071**	**1055**	**1049**	**1071**	**1052**	**1104**	**1105**	**1109**
L	1309	1287	1341	1289	1261	1247	1246	1238	1280	1247	1238	1278	1174	1180	1172
Lunamb	4615	4615	4615	4615	4615	4615	4615	4615	4615	4615	4615	4615	4615	4615	4615
Lcomb	5945	5915	5976	5941	5909	5895	5885	5886	5939	5886	5886	5927	5808	5817	5804
ILD$_{index}$	0.00353	0.00219*	0.00334	0.00622	0.00558	0.00559	0.00407	0.00560	0.00740	0.00407	0.00560	0.00573	0.00327	0.00378	0.00292
R-ILD$_{index}$	0.40384	0.17567	1	0.51388	0.33	0.28947	0.20869	0.26829	0.54320	0.21052	0.26829	0.40963	0.10160	0.12154	0.08994*
**ITS2**															
Chars	**900**	**901**	**908**	**890**	**887**	**902**	**885**	**887**	**891**	**890**	**889**	**896**	**925**	**922**	**928**
L	1329	1326	1296	1299	1295	1295	1336	1331	1298	1323	1317	1302	1249	1246	1259
Lunamb	4385	4385	4385	4385	4385	4385	4385	4385	4385	4385	4385	4385	4385	4385	4385
Lcomb	5739	5736	5712	5706	5701	5711	5745	5734	5715	5733	5724	5708	5648	5649	5656
ILD$_{index}$	0.00435	0.00435	0.00542	0.00385	0.00368	0.00542	0.00417	0.00313	0.00559	0.00436	0.00384	0.00367	0.00247	0.00318	0.00212*
R-ILD$_{index}$	0.80645	0.73529	0.48437	0.36065	0.32307	0.47692	1	0.62068	0.51612	0.67567	0.51162	0.36206	0.12612	0.15789	0.11881*
**HUWE1**															
Chars	**655**	**654**	**654**	**656**	**657**	**657**	**656**	**657**	**657**	**656**	**657**	**657**	**658**	**658**	**658**
L	353	357	357	355	354	352	355	354	352	355	354	350	353	353	350
Lunamb	3801	3801	3801	3801	3801	3801	3801	3801	3801	3801	3801	3801	3801	3801	3801
Lcomb	4168	4168	4168	4172	4170	4167	4172	4170	4167	4172	4170	4165	4165	4165	4162
ILD$_{index}$	0.00335	0.00239*	0.00239*	0.00383	0.00359	0.00335	0.00383	0.00359	0.00335	0.00383	0.00359	0.00336	0.00264	0.00264	0.00264
R-ILD$_{index}$	0.77777	0.71428	0.71428	1	0.88235	0.73684	1	0.88235	0.73684	1	0.88235	0.66666	0.61111	0.61111	0.52380*

Chars: total number of characters; *L*: tree length of each individual fragment tree; *Lcomb*: tree length when combined with unambiguously aligned regions; *Lunamb*: tree length of unambiguously aligned regions; *ILD*$_{index}$=ILD/ Lcomb; *R-ILD*$_{index}$=ILD/(Lcomb$_{max}$-(L+Lunamb). ILD=Lcomb-(L+Lunamb)

Tabla S4. Marginal likelihood values in BEAST analyses for individual gene fragments as estimated in Tracer v1.5. Selected analyses are shown in bold. **a)** Individual gene fragments with *Ingroup* datasets. **b)** Individual gene fragments with *Outgroup* datasets. **c)** Combined datasets.

a)

Matrix	Analytical conditions	MargL	ΔP	Add. BF	R-MargL
cox1-A	NP_SC	-4339.84	0	0	-4339.84
	NP_ULN	-4337.71	1	10	-4347.71
	**2P_SC**	**-4043.74**	**10**	**100**	**-4143.74**
	2P_ULN	-4042.4	11	110	-4152.4
	3P_SC	-3996.42	20	200	-4196.42
	3P_ULN	-3994.52	21	210	-4204.52
cox1-B	NP_SC	-5261.66	0	0	-5261.66
	NP_ULN	-5260.47	1	10	-5270.47
	**2P_SC**	**-4899.29**	**10**	**100**	**-4999.29**
	2P_ULN	-4899.15	11	110	-5009.15
	3P_SC	-4874.37	20	200	-5074.37
	3P_ULN	-4874.58	21	210	-5084.58
cob	NP_SC	-4967.52	0	0	-4967.52
	NP_ULN	-4963.01	1	10	-4973.01
	**2P_SC**	**-4644.97**	**10**	**100**	**-4744.97**
	2P_ULN	-4641.57	11	110	-4751.57
	3P_SC	-4621.3	20	200	-4821.3
	3P_ULN	-4616.92	21	210	-4826.92
nd5	NP_SC	-5539.99	0	0	-5539.99
	NP_ULN	-5539.2	1	10	-5549.2
	**2P_SC**	**-5241.01**	**10**	**100**	**-5341.01**
	2P_ULN	-5240.49	11	110	-5350.49
	3P_SC	-5218.69	20	200	-5418.69
	3P_ULN	-5217.58	21	210	-5427.58
***HUWE1* (only coding)**	NP_SC	-2098.58	0	0	-2098.58
	**NP_ULN**	**-2086.67**	**1**	**10**	**-2096.67**
	2P_SC	-2016.86	10	100	-2116.86
	2P_ULN	-2002.78	11	110	-2112.78
	3P_SC	-2015.17	20	200	-2215.17
	3P_ULN	-2001.59	21	210	-2211.59
rrnl	**Nogaps_SC**	**-2218.2**	**0**	**0**	**-2218.2**
	Nogaps_ULN	-2211.6	1	10	-2221.6
	**Allgaps_SC**	**-2332.54**	**0**	**0**	**-2332.54**
	Allgaps_ULN	-2324.95	1	10	-2334.95
	**Complete_SC**	**-2332.54**	**0**	**0**	**-2332.54**
	Complete_ULN	-2324.95	1	10	-2334.95
LSU-A	**Nogaps_SC**	**-2126.15**	**0**	**0**	**-2126.15**
	Nogaps_ULN	-2119.52	1	10	-2129.52
	Allgaps_SC	-2319.44	0	0	-2319.44
	**Allgaps_ULN**	**-2306.35**	**1**	**10**	**-2316.35**
	Complete_SC	-2324.56	0	0	-2324.56
	**Complete_ULN**	**-2310.82**	**1**	**10**	**-2320.82**
LSU-B	Nogaps_SC	-1328.58	0	0	-1328.58
	**Nogaps_ULN**	**-1315.57**	**1**	**10**	**-1325.57**
	Allgaps_SC	-4511.57	0	0	-4511.57
	**Allgaps_ULN**	**-4480.6**	**1**	**10**	**-4490.6**
	Complete_SC	-5049.25	0	0	-5049.25
	**Complete_ULN**	**-5017.46**	**1**	**10**	**-5027.46**
ITS2	**Nogaps_SC**	**-1295.63**	**0**	**0**	**-1295.63**
	Nogaps_ULN	-1287.35	1	10	-1297.35
	Allgaps_SC	-3926	0	0	-3926
	**Allgaps_ULN**	**-3903.52**	**1**	**10**	**-3913.52**
	Complete_SC	-4557.93	0	0	-4557.93
	**Complete_ULN**	**-4537.43**	**1**	**10**	**-4547.43**
***HUWE1* (complete)**	**Complete_SC**	**-2578.58**	**0**	**0**	**-2578.58**
	Complete_ULN	-2571.02	1	10	-2581.02

b)

Matrix	Analytical conditions	MargL	ΔP	Add. BF	R-MargL
cox1-A	NP_SC	-5517.38	0	0	-5517.38
	NP_ULN	-5509.7	1	10	-5519.7
	**2P_SC**	**-5172**	**10**	**100**	**-5272**
	2P_ULN	-5167.37	11	110	-5277.37
	3P_SC	-5110.42	20	200	-5310.42
	3P_ULN	-5105.81	21	210	-5315.81
cox1-B	NP_SC	-6675.26	0	0	-6675.26
	NP_ULN	-6674.23	1	10	-6684.23
	**2P_SC**	**-6268.86**	**10**	**100**	**-6368.86**
	2P_ULN	-6268.34	11	110	-6378.34
	3P_SC	-6231.2	20	200	-6431.2
	3P_ULN	-6230.5	21	210	-6440.5
cob	NP_SC	-6253.32	0	0	-6253.32
	NP_ULN	-6248.12	1	10	-6258.12
	**2P_SC**	**-5914.03**	**10**	**100**	**-6014.03**
	2P_ULN	-5910.52	11	110	-6020.52
	3P_SC	-5886.2	20	200	-6086.2
	3P_ULN	-5882.79	21	210	-6092.79
nd5	NP_SC	-6732.97	0	0	-6732.97
	NP_ULN	-6731.72	1	10	-6741.72
	**2P_SC**	**-6399.25**	**10**	**100**	**-6499.25**
	2P_ULN	-6398.35	11	110	-6508.35
	3P_SC	-6364.12	20	200	-6564.12
	3P_ULN	-6363.38	21	210	-6573.38
HUWE1 **(only coding)**	NP_SC	-2434.21	0	0	-2434.21
	**NP_ULN**	**-2405.52**	**1**	**10**	**-2415.52**
	2P_SC	-2337	10	100	-2437
	2P_ULN	-2307.88	11	110	-2417.88
	3P_SC	-2348.03	20	200	-2548.03
	3P_ULN	-2321.11	21	210	-2531.11
rrnl	**Nogaps_SC**	**-2757.36**	**0**	**0**	**-2757.36**
	Nogaps_ULN	-2747.8	1	10	-2757.8
	Allgaps_SC	-3126.82	0	0	-3126.82
	**Allgaps_ULN**	**-3115.34**	**1**	**10**	**-3125.34**
	Complete_SC	-3126.82	0	0	-3126.82
	**Complete_ULN**	**-3115.34**	**1**	**10**	**-3125.34**
LSU-A	Nogaps_SC	-3173.41	0	0	-3173.41
	**Nogaps_ULN**	**-3154.55**	**1**	**10**	**-3164.55**
	Allgaps_SC	-4413.07	0	0	-4413.07
	**Allgaps_ULN**	**-4387.9**	**1**	**10**	**-4397.9**
	Complete_SC	-4587.12	0	0	-4587.12
	**Complete_ULN**	**-4561.94**	**1**	**10**	**-4571.94**
LSU-B	Nogaps_SC	-1586.17	0	0	-1586.17
	**Nogaps_ULN**	**-1572.03**	**1**	**10**	**-1582.03**
	Allgaps_SC	-4826.68	0	0	-4826.68
	**Allgaps_ULN**	**-4787.38**	**1**	**10**	**-4797.38**
	Complete_SC	-6393.2	0	0	-6393.2
	**Complete_ULN**	**-6348.57**	**1**	**10**	**-6358.57**
ITS2	Nogaps_SC	-332.82	0	0	-332.82
	**Nogaps_ULN**	**-310.21**	**1**	**10**	**-320.21**
	Allgaps_SC	-5045.99	0	0	-5045.99
	**Allgaps_ULN**	**-4999.22**	**1**	**10**	**-5009.22**
	Complete_SC	-7587.44	0	0	-7587.44
	**Complete_ULN**	**-7547**	**1**	**10**	**-7557**
HUWE1 **(complete)**	Complete_SC	-3002.51	0	0	-3002.51
	**Complete_ULN**	**-2974.29**	**1**	**10**	**-2984.29**

c)

Matrix	dataset	Strategie	MargL	ΔP	Add. BF	R-MargL
**MIT**	Ingroup	SC	-22768.03	0	0	-22768.03
		ULN	-22758.92	1	10	-22768.92
		G-NP_SC	-22520.67	50	500	-23020.67
		G-NP_ULN	-22508.43	51	510	-23018.43
		**G-2P_SC**	**-21248.89**	**90**	**900**	**-22148.89**
		G-2P_ULN	-21240.4	91	910	-22150.4
		G-3P_SC	-21127.42	130	1300	-22427.42
		G-3P_ULN	-21117.83	131	1310	-22427.83
**MIT**	With outgroups	SC	-28407.07	0	0	-28407.07
		ULN	-28399.12	1	10	-28409.12
		G-NP_SC	-28085.3	50	500	-28585.3
		G-NP_ULN	-28076.09	51	510	-28586.09
		**G-2P_SC**	**-26667.96**	**90**	**900**	**-27567.96**
		G-2P_ULN	-26663.08	91	910	-27573.08
		G-3P_SC	-26503.97	130	1300	-27803.97
		G-3P_ULN	-26498.05	131	1310	-27808.05
**MIT_NUC**	Ingroup	SC	-37444.87	0	0	-37444.87
		ULN	-37409.85	1	10	-37419.85
		G-NP_SC	-36551.33	90	900	-37451.33
		G-NP_ULN	-36507.06	91	910	-37417.06
		G-2P_SC	-35323.69	130	1300	-36623.69
		**G-2P_ULN**	**-35276.92**	**131**	**1310**	**-36586.92**
		G-3P_SC	-35200.47	170	1700	-36900.47
		G-3P_ULN	-35154.73	171	1710	-36864.73
**MIT_NUC**	With outgroups	SC	-48072.62	0	0	-48072.62
		ULN	-48036.54	1	10	-48046.54
		G-NP_SC	-46768.25	90	900	-47668.25
		G-NP_ULN	-46730.3	91	910	-47640.3
		G-2P_SC	-45423.25	130	1300	-46723.25
		**G-2P_ULN**	**-45385.39**	**131**	**1310**	**-46695.39**
		G-3P_SC	-45256.11	170	1700	-46956.11
		G-3P_ULN	-45218.19	171	1710	-46928.19
**NUC**	Ingroup	SC	-13773.02	0	0	-13773.02
		**ULN**	**-13721.01**	**1**	**10**	**-13731.01**
		G-NP_SC	-13658.64	40	400	-14058.64
		G-NP_ULN	-13600.84	41	410	-14010.84
**NUC**	With outgroups	SC	-17812.02	0	0	-17812.02
		**ULN**	**-17738.64**	**1**	**10**	**-17748.64**
		G-NP_SC	-17637.36	40	400	-18037.36
		G-NP_ULN	-17558.39	41	410	-17968.39

MargL. Ln Marginal Likelihoods as estimated in Tracer v.1.5.

ΔP. Additional parameters with respect to the strategy showing the small number of model parameters.

Add. BF. Adding LnBF Value**:** Necessary improvement in Ln Marginal Likelihood to be favored, considering the requirement of 10 LnBF unities per additional parameter

R-MargL. Reescaled Ln Marginal Likelihoods (Marginal Likelihood + Adding LnBF Value) that allow for direct comparisons of nested analyses, the higher R-MargL is favoured.

Table S5. Mean rates of molecular evolution and 95% HPD intervals in calibration analyses on the genus *Carabus* for: a) Protein coding genes. b) Non coding genes. c) Combined datasets. O.G. *Outgroup* dataset; I.G. *Ingroup* dataset

a)

		NP_SC	NP_ULN	2P_SC	2P_ULN	3P_SC	3P_ULN
***cox1*-A**	**I.G.**	0.0092 (0.0066-0.0118)	0.0092 (0.0066-0.012)	0.0113 (0.0081-0.0147)	0.0114 (0.0081-0.0151)	0.0114 (0.0082-0.015)	0.0115 (0.0081-0.0152)
	**O.G.**	0.0103 (0.0073-0.0136)	0.011 (0.0075-0.015)	0.0118 (0.0085-0.0157)	0.0122 (0.0084-0.0163)	0.012 (0.0084-0.0159)	0.0122 (0.0085-0.0164)
***cox1*-B**	**I.G.**	0.011 (0.0076-0.0147)	0.0111 (0.0076-0.0148)	0.0145 (0.01-0.0198)	0.0148 (0.0098-0.02)	0.015 (0.0103-0.0208)	0.0152 (0.0103-0.021)
	**O.G.**	0.0139 (0.009-0.0194)	0.0139 (0.009-0.0194)	0.0178 (0.0118-0.0247)	0.0179 (0.0116-0.025)	0.0189 (0.0123-0.0265)	0.019 (0.0122-0.0268)
cob	**I.G.**	0.0179 (0.0102-0.0274)	0.0184 (0.0102-0.0289)	0.0251 (0.0151-0.0369)	0.0258 (0.0148-0.0388)	0.0264 (0.0155-0.0394)	0.0273 (0.0156-0.0418)
	**O.G.**	0.0237 (0.0141-0.0351)	0.0257 (0.0138-0.0388)	0.0314 (0.018-0.0485)	0.0337 (0.0175-0.0531)	0.0345 (0.018-0.0542)	0.0366 (0.0191-0.0592)
nd5	**I.G.**	0.0159 (0.0102-0.0223)	0.0159 (0.0102-0.0224)	0.0273 (0.0151-0.0426)	0.0272 (0.0148-0.0422)	0.0308 (0.0163-0.0489)	0.0315 (0.0166-0.0503)
	**O.G.**	0.0172 (0.0116-0.0235)	0.0173 (0.0115-0.0236)	0.0443 (0.0222-0.0719)	0.0446 (0.0223-0.0721)	0.0524 (0.0256-0.0855)	0.0529 (0.0249-0.0859)
**HUWE1**	**I.G.**	0.0021 (0.0015-0.0028)	0.0023 (0.0015-0.0033)	0.0021 (0.0015-0.0027)	0.0024 (0.0015-0.0033)	0.0021 (0.0015-0.0027)	0.0024 (0.0015-0.0033)
(only coding)	**O.G.**	0.0021 (0.0015-0.0028)	0.0026 (0.0016-0.0037)	0.0021 (0.0015-0.0027)	0.0027 (0.0016-0.0038)	0.0021 (0.0015-0.0027)	0.0027 (0.0016-0.0038)

b)

		Nogaps_SC	Nogaps_ULN	Allgaps_SC	Allgaps_ULN	Complete_SC	Complete_ULN
rrnl	**I.G.**	0.0017 (0.0011-0.0024)	0.0018 (0.0011-0.0026)	0.0016 (0.001-0.0022)	0.0018 (0.0011-0.0026)	0.0016 (0.001-0.0022)	0.0018 (0.0011-0.0026)
	**O.G.**	0.0015 (0.001-0.002)	0.0018 (0.001-0.0027)	0.0016 (0.0011-0.0022)	0.0019 (0.0011-0.0028)	0.0016 (0.0011-0.0022)	0.0019 (0.0011-0.0028)
LSU-A	**I.G.**	0.0009 (0.0006-0.0013)	0.0011 (0.0006-0.0017)	0.001 (0.0007-0.0014)	0.0013 (0.0007-0.002)	0.001 (0.0006-0.0014)	0.0013 (0.0007-0.002)
	**O.G.**	0.0009 (0.0006-0.0013)	0.0043 (0.0018-0.0078)	0.0012 (0.0008-0.0016)	0.0051 (0.002-0.0091)	0.0011 (0.0008-0.0015)	0.0051 (0.002-0.0089)
***LSU*-B**	**I.G.**	0.0009 (0.0004-0.0014)	0.0012 (0.0005-0.002)	0.0034 (0.0025-0.0044)	0.0064 (0.0037-0.0094)	0.004 (0.003-0.0052)	0.0072 (0.0043-0.0105)
	**O.G.**	0.0009 (0.0005-0.0014)	0.003 (0.0014-0.005)	0.0022 (0.0016-0.0029)	0.0058 (0.0028-0.0091)	0.0037 (0.0028-0.0048)	0.0102 (0.0045-0.0165)
ITS2	**I.G.**	0.0017 (0.0011-0.0025)	0.0024 (0.0012-0.0038)	0.0038 (0.0029-0.0049)	0.0057 (0.0035-0.0081)	0.0043 (0.0031-0.0054)	0.0063 (0.0039-0.0091)
	**O.G.**	0.0029 (0.0012-0.0049)	0.0082 (0.0023-0.0163)	0.003 (0.0022-0.0039)	0.0104 (0.0055-0.0159)	0.0044 (0.0033-0.0055)	0.0157 (0.0073-0.0249)
**HUWE1**	**I.G.**	n/a	n/a	n/a	n/a	0.0021 (0.0015-0.0027)	0.0022 (0.0015-0.0029)
	**O.G.**	n/a	n/a	n/a	n/a	0.0021 (0.0016-0.0027)	0.0024 (0.0016-0.0033)

c)

		NP-SC	NP-ULN	G-NP_SC	G-NP_ULN	G-2P_SC	G-2P_ULN	G-3P_SC	G-3P_ULN
**MIT**	**I.G.**	0.0087 (0.0071-0.0104)	0.0088 (0.007-0.0106)	0.0101 (0.0082-0.0121)	0.0102 (0.0081-0.0123)	0.0134 (0.0108-0.0162)	0.0136 (0.0106-0.0166)	0.0137 (0.011-0.0168)	0.014 (0.011-0.0173)
**MIT**	**O.G.**	0.0103 (0.0083-0.0124)	0.0102 (0.0082-0.0125)	0.012 (0.0096-0.0146)	0.0119 (0.0093-0.0145)	0.0156 (0.0124-0.0191)	0.0156 (0.0123-0.0192)	0.0164 (0.0129-0.0201)	0.0164 (0.0127-0.0203)
**NUC**	**I.G.**	0.0023 (0.0019-0.0027)	0.0029 (0.002-0.0039)	0.0029 (0.0023-0.0035)	0.0038 (0.0026-0.005)	n/a	n/a	n/a	n/a
**NUC**	**O.G.**	0.0019 (0.0015-0.0023)	0.0044 (0.0024-0.0065)	0.0022 (0.0017-0.0026)	0.0064 (0.0033-0.0099)	n/a	n/a	n/a	n/a
**MIT-NUC**	**I.G.**	0.0045 (0.0038-0.0052)	0.0047 (0.0039-0.0056)	0.0061 (0.005-0.0071)	0.0064 (0.0052-0.0077)	0.0075 (0.0063-0.0088)	0.0080 (0.0064-0.0097)	0.0075 (0.0063-0.0088)	0.0081 (0.0064-0.0098)
**MIT-NUC**	**O.G.**	0.0044 (0.0037-0.0051)	0.0047 (0.0039-0.0056)	0.005 (0.0042-0.0059)	0.0056 (0.0045-0.0067)	0.006 (0.005-0.007)	0.0067 (0.0054-0.0082)	0.006 (0.0051-0.0071)	0.0067 (0.0054-0.0082)

Table S6. Mean ages and 95% HPD interval in calibration analyses on the genus *Carabus* for: a) Protein coding gene. b) Non coding genes. c) Combined datasets. O.G. *Outgroup* dataset; I.G. *Ingroup* dataset

a)

		**NP_SC**	**NP_ULN**	**2P_SC**	**2P_ULN**	**3P_SC**	**3P_ULN**
***cox1*-A**	I.G.	19.52 (15.08-24.97)	19.57 (14.64-25.57)	19.79 (15.2-24.9)	20.03 (15.13-26.05)	19.8 (15.34-25.02)	20 (14.95-25.79)
	O.G.	20.04 (15.43-25.05)	21.12 (15.32-28.37)	19.95 (15.32-25.3)	21.04 (15.43-28.22)	20.37 (15.68-26.1)	21.16 (15.76-27.95)
***cox1*-B**	I.G.	19.65 (15.19-25.02)	19.42 (14.85-24.89)	21.58 (16.43-27.71)	21.41 (16.01-27.69)	21.78 (16.47-28.01)	21.63 (16.26-28.25)
	O.G.	23.07 (17.54-29.14)	23.46 (17.64-30.18)	24.42 (18.76-31.41)	24.99 (18.56-32.48)	25.06 (18.91-32.12)	25.71 (19.17-33.74)
cob	I.G.	23.56 (17.64-30.1)	24.28 (17.31-32.82)	25.77 (19.75-32.91)	26.42 (19.01-35.8)	25.91 (19.79-33.11)	26.48 (19.05-35.69)
	O.G.	26.33 (19.66-33.97)	27.3 (19.2-36.95)	27.23 (20.68-35.12)	28.24 (20.15-38.64)	27.43 (20.93-35.37)	28.53 (20.42-38.61)
nd5	I.G.	20.71 (15.9-26.15)	20.66 (15.64-26.69)	21.57 (16.72-27.39)	21.54 (16.17-27.51)	21.85 (16.84-27.7)	21.83 (16.44-28.06)
	O.G.	21.43 (16.8-26.77)	21.48 (16.54-27.15)	22.8 (17.51-28.69)	22.8 (17.27-29.05)	23.36 (18.01-29.6)	23.3 (17.66-30.01)
**HUWE1**	I.G.	31.08 (21.64-43.3)	23.89 (14.41-37.6)	31.56 (21.46-43.36)	24.04 (14.83-39.01)	31.27 (21.49-43.31)	23.99 (14.17-38)
(only coding)	O.G.	27.1 (19.04-38.69)	25.33 (14.83-40.82)	27.35 (18.63-38.4)	24.67 (14.23-39.51)	27.09 (19.08-37.96)	24.77 (14.36-39.95)

b)

		Nogaps_SC	Nogaps_ULN	Allgaps_SC	Allgaps_ULN	Complete_SC	Complete_ULN
rrnl	I.G.	25.5 (16.23-37.05)	24.19 (14.6-37.43)	29.91 (19.4-42.76)	26.91 (16.11-42.01)	29.91 (19.4-42.76)	26.91 (16.11-42.01)
	O.G.	24.93 (16.31-35.38)	24.82 (15.03-37.21)	28.78 (19.52-41.07)	26.52 (16.58-40.45)	28.78 (19.52-41.07)	26.52 (16.58-40.45)
***LSU*-A**	I.G.	23.99 (14.72-35.97)	13.31 (8.29-25.74)	23.92 (14.76-35.8)	13.37 (8.35-24.85)	23.83 (14.37-35.79)	13.29 (8.35-25.06)
	O.G.	28.7 (16.96-44.16)	15.44 (8.28-29.15)	23.19 (14.16-34.74)	17.44 (8.89-33.61)	23.26 (14.44-34.55)	17.99 (8.74-35.34)
***LSU*-B**	I.G.	23.65 (10.96-40)	11.46 (7.95-20.74)	59.33 (42.02-80.87)	20.36 (11.23-36.61)	59.32 (42.1-80.63)	22.35 (12.14-39.68)
	O.G.	27 (13.73-44.62)	10.41 (7.87-16.29)	88.14 (60.72-121.0)	26.2 (12.64-50.29)	86.09 (60.79-115.8)	27.72 (13.14-52.09)
ITS2	I.G.	52.43 (30.67-79.83)	30.56 (14.93-54.35)	60.85 (44.15-80.42)	31.17 (16.8-52.47)	63.69 (46.12-83.92)	33.83 (18.25-57.46)
	O.G.	9.94 (7.61-14.46)	11.21 (7.99-18.14)	45.23 (32.31-60.79)	29.76 (15.99-51.55)	58.02 (43.36-75.77)	36.06 (16.86-64.47)
**HUWE1**	I.G.	n/a	n/a	n/a	n/a	30.83 (21.79-41.36)	25.91 (16.57-39.3)
	O.G.	n/a	n/a	n/a	n/a	26.23 (19.24-35.03)	27.16 (16.13-41.91)

c)

		NP_SC	NP_ULN	G-NP_SC	G-NP_ULN	G-2P_SC	G-2P_ULN	G-3P_SC	G3P_ULN
**MIT**	**I.G.**	19.9 (16.6-23.25)	20.04 (16.23-24.28)	19.91 (16.75-23.39)	20.04 (16.01-24.28)	21.58 (17.98-25.4)	21.76 (17.62-26.87)	21.68 (18.11-25.57)	21.8 (17.49-26.59)
**MIT**	**O.G.**	20.65 (17.27-24.1)	20.94 (17.37-25.03)	20.41 (17.03-23.82)	20.74 (16.9-24.59)	21.21 (17.77-24.95)	21.32 (17.57-25.19)	21.42 (17.89-25.03)	21.56 (17.84-25.72)
**NUC**	**I.G.**	43.19 (34.72-52.41)	28.5 (16.97-44.65)	43.47 (34.66-52.87)	28.2 (16.6-45.05)	n/a	n/a	n/a	n/a
**NUC**	**O.G.**	39.08 (31.45-47.88)	29.25 (17.6-44.66)	39.27 (31.53-48.02)	28.31 (16.63-44.63)	n/a	n/a	n/a	n/a
**MIT-NUC**	**I.G.**	22.3 (18.79-25.9)	21.48 (16.49-27.02)	25.11 (20.94-29.23)	23.84 (17.89-31.17)	26.75 (22.31-31.33)	25.16 (18.41-33.04)	26.81 (22.52-31.46)	25.14 (18.6-33.38)
**MIT-NUC**	**O.G.**	20.98 (17.82-24.36)	20.84 (17.18-25.01)	23.45 (19.8-27.29)	22.67 (18.46-27.55)	24.13 (20.44-28.32)	23.07 (18.79-28.22)	24.76 (20.8-29.04)	24.06 (19.1-29.47)

C) Supplementary text

Text S1.Calibration hypotheses used for dating of genus *Carabus*.

1. The origin of Gran Canaria 14.5 Mya (Hoernle et al. 1991) was used as a conservative maximum age for the split between two Canarian endemic species, *Carabus (Nesaeocarabus) coarctatus* from Gran Canaria and its sister *C. (N.) abbreviatus* from Tenerife [node C] (Prüser et al. 2000).

2. The origin of *C. (Autocarabus) cancellatus* can be given a minimum age of 5.3 Ma as estimated by Deuve (1988) for a fossil assigned to this species found in Murat (Cantal, France). This is implemented on the phylogeny to the split of this species with its sister *C. auratus* [node F].

3. The last land connections between Japan and the continent occurred 3.5 Mya (Iijima and Tada 1990; Koizumi 1992; Tada 1994; Koizumi et al. 2009), although major climatic and geological changes during the Pleistocene (Kitamura *et al.* 2001; Taira 2001) may have provided a later chance for dispersal of terrestrial fauna. The diversification of the endemic *Carabus (Damaster) blaptoides* species complex [node J1], and those of the subgenera *Ohomopterus* [node J2] and *Leptocarabus* [node J3] were assumed to have occurred during this period (Nagata, et al. 2007). The split of *Ohomopterus* and continental relatives, such as *Isiocarabus* [node J4b] (Tominaga et al. 2000) was linked to the initial tectonic separation of the Japanese archipelago from the mainland around 15 Mya (Jolivet et al. 1994; Otofuji *et al.* 1994; Otofuji 1996).

4. The Messinian geologic events that occurred in the western Mediterranean were arguably responsible for many taxonomic splits and the observed current distribution patterns for many organisms. The opening of the Gibraltar Strait at 5.33 Ma marked the end of the Messinian, and the rapid refilling of the Mediterranean basin (Krijgsman *et al.* 1999; García-Castellanos *et al.* 2009) isolated emerged areas and provided a solid basis for molecular calibrations. Two phylogenetic patterns in *Carabus* have been seen to fit this scenario (Andújar et al. in press): the separation between *Carabus (Mesocarabus) riffensis* and Iberian *Mesocarabus* [node M1] and the split between *C. (Eurycarabus) genei* from Sardinia and North African *Eurycarabus* [node M2] (Prüser and Mossakowski 1998).

D) Supplementary References

Arndt E, Brücker M, Marciniak K, Mossakowski D, Prüser F: 2003. **Phylogeny**. In *The genus Carabus L. in Europe, a synthesis.* Edited by H. Turin & L. Penev. Sofía-Moscow-Leiden: PENSOFT & European Invertebrate Survey; 2003:307-325.

Crozier RH, Crozier YC: **The cytochrome-b and ATPase genes of honeybee mitochondrial-DNA.** *Mol Biol Evol* 1992, **9:**474-482.

Deuve T: **Three remarkably well-preserved fossil insects from the Miocene of France, belonging to the genera *Carabus* L. and *Ledouxnebria* nov. (Coleoptera, Carabidae and Nebriidae).** *Bull Soc Entomol Fr* 1988, **103:** 229-236.

Düring A, Brücker M: **The evolutionary history of the tribe Molopini: a first molecular approach.** In *Proceedings of the 9th international carabidologist meeting*. Edited by P. Brandmayer, GL. Lövei, Z. Brandmayer, A. Casale & V. Taglianti. Sofia, Pensoft publisher, 2000:1-4.

Folmer O, Black M, Hoeh W, Lutz R, Vrijenhoek R: **DNA primers for amplification of mitochondrial cytochrome c oxidase subunit I from diverse metazoan invertebrates.** *Mol Marine Biol Biotech* 1994, **3:** 294-299.

García-Castellanos D, Estrada F, Jiménez-Munt I, Gorini C, Fernández M, Verges J, De Vicente R: **Catastrophic flood of the Mediterranean after the Messinian salinity crisis.** *Nature* 2009, **462:**778-U96.

Harry M, Solignac M, Lachaise D: **Molecular evidence for parallel evolution of adaptive syndromes in fig-breeding Lissocephala (Drosophilidae).** *Mol Phylogenet Evol* 1998, **9:**542-551.

Hoernle K, Tilton G, Schmincke HU: **Sr-Nd-Pb isotopic evolution of Gran-Canaria - Evidence for shallow enriched mantle beneath the Canary-Islands.** *Earth Planet Sci Lett* 1991, **106:**44-63.

Iijima A, Tada R: **Evolution of Tertiary sedimentary basins of Japan in reference to opening of the Japan Sea.** *J of the Faculty of Science, Univ of Tokyo* 1990, **22:** 121-171.

Jolivet L, Tamaki K, Fournier M: **Japan sea, opening history and mechanism - A synthesis.** *J Geophys res-Sol Ea* 1994, **99:**22237-22259.

Kim CG, Su Z-H, Imura Y, Okamoto M, Osawa S: **Phylogeny and evolution of the division Procrustimorphi (Coleoptera, Carabidae) of the world as deduced from the mitochondrial ND5 gene sequences.** *Elytra Tokyo* 2003, **31:**263-284.

Kim CG, Tominaga O, Su Z.H, Osawa S: **Differentiation within the genus *Leptocarabus* (excl. *L. kurilensis*) in the Japanese Islands as deduced from mitochondrial ND5 gene sequences (Coleoptera, Carabidae).** *Genes Genet Syst* 2000a, **75:**335-342.

Kim CG, Zhou H.Z, Imura Y, Tominaga O, Su Z.H, Osawa S: **Pattern of morphological diversification in the *Leptocarabus* ground beetles (Coleoptera: Carabidae) as deduced

from mitochondrial ND5 gene and nuclear 28S rDNA sequences. *Mol Biol Evol* 2000b, **17:**137-145.

Kitamura A, Takano O, Takata H, Omote H: **Late Pliocene-early Pleistocene paleoceanographic evolution of the Sea of Japan.** *Palaeogeog palaeoclim palaeoecol* 2001, **172:**81-98.

Koizumi I, Sato M, Matoba Y: **Age and significance of Miocene diatoms and diatomaceous sediments from northeast Japan.** *Palaeogeog palaeoclim palaeoecol* 2009, **272:**85-98.

Koizumi I: **Diatom biostratigraphy of the Japan Sea.** In *Proc. ODP. Sci. Results, vol.127/128.* Edited by Pisciotto et al. College Station, TX:Ocean Drilling Program. 1992:249-289.

Krijgsman W, Hilgen FJ, Raffi I, Sierro FJ, Wilson DS: **Chronology, causes and progression of the Messinian salinity crisis.** *Nature* 1999, **400:**652-655.

Nagata N, Kubota K, Sota T: **Phylogeography and introgressive hybridization of the ground beetle *Carabus yamato* in Japan based on mitochondrial gene sequences.** *Zool Sci* 2007, **24:**465-474.

Ober KA: **Phylogenetic relationships of the carabid subfamily Harpalinae (Coleoptera) based on molecular sequence data.** *Mol Phylogenet Evol* 2002, **24:**228-248.

Okamoto M, Kashiwai N, Su ZH, Osawa S: **Sympatric convergence of the color pattern in the Chilean *Ceroglossus* ground beetles inferred from sequence comparisons of the mitochondrial ND5 gene**. *J Mol Evol* 2001, **53:**530-538.

Otofuji Y, Kambara A, Matsuda T, Nohda S: **Counterclockwise rotation of Northeast Japan - Paleomagnetic evidence for regional extent and timing of rotation.** *Earth Planet Sci Lett* 1994, **121:**503-518.

Otofuji Y: **Large tectonic movement of the Japan Arc in late Cenozoic times inferred from paleomagnetism: Review and synthesis.** *Island Arc* 1996, **5:**229-249.

Porter CH, Collins FH: **Species-diagnostic differences in a ribosomal DNA internal transcribed spacer from the sibling species Anopheles-freeborni and Anopheles-hermsi (Diptera, Culicidae).** *Am J Trop Med Hyg* 1991, 45, 271-279.

Prüser F, Brücker M, Mossakowski D: **Colonization of Canary Islands by *Carabus* species: evidence from different character complexes.** In *Proceedings of the 9th international carabidologist meeting.* Edited by P. Brandmayer, GL. Lövei, Z. Brandmayer, A. Casale & V. Taglianti. Sofia, Pensoft publisher, 2000:45-52.

Pruser F, Mossakowski D: **Low substitution rates in mitochondrial DNA in Mediterranean carabid beetles.** *Insect Mol Biol* 1998, **7:**121-128.

Simon C, Frati F, Beckenbach A, Crespi B, Liu H, Flook P: **Evolution, weighting, and phylogenetic utility of mitochondrial gene-sequences and a compilation of conserved Polymerase Chain-Reaction primers.** *Ann Entomol Soc Am* 1994, **87:**651-701.

Sota T, Vogler AP: **Incongruence of mitochondrial and nuclear gene trees in the carabid beetles *Ohomopterus*.** *Syst Biol* 2001, **50:**39-59.

Sota T, Takami Y, Monteith GB, Moore BP: **Phylogeny and character evolution of endemic Australian carabid beetles of the genus *Pamborus* based on mitochondrial and nuclear gene sequences.** *Mol Phylogenet Evol* 2005, **36:**391-404.

Su ZH, Ohama T, Okada TS, Nakamura K, Ishikawa R, Osawa S: **Phylogenetic relationships and evolution of the Japanese carabinae ground beetles based on mitochondrial ND5 gene sequences.** *J Mol Evol* 1996a, **42:**124-129.

Su ZH, Okada TS, Osawa S, David B, Dommergues JL, Magniez F: **Radiation of several Carabina groups (Coleoptera, Carabidae) inferred from the mitochondrial ND5 gene sequences.** *Elytra Tokyo* 1996b, **24:**175-179.

Su ZH, Tominaga O, Okamoto M, Osawa S: **Origin and diversification of hindwingless Damaster ground beetles within the Japanese islands as deduced from mitochondrial ND5 gene sequences (Coleoptera, Carabidae).** *Mol Biol Evol* 1998, **15:**1026-1039.

Su ZH, Imura Y, Zhou HZ, Okamoto M, Osawa S: **Mode of morphological differentiation in the Latitarsi-ground beetles (Coleoptera, Carabidae) of the world inferred from a phylogenetic tree of mitochondrial ND5 gene sequences.** *Genes Genet Syst* 2003, **78:**53-70.

Su ZH, Imura Y, Okamoto M, Osawa S: **Pattern of phylogenetic diversification of the Cychrini ground beetles in the world as deduced mainly from sequence comparisons of the mitochondrial genes.** *Gene* 2004, **326:**43-57.

Tada R: **Paleoceanographic evolution of the Japan Sea**. *Palaeogeog palaeoclim palaeoecol* 1994, **108:**487-508.

Taira A: **Tectonic evolution of the Japanese island arc system.** *Annu Rev Earth Planet Sci* 2001, **29:**109-134.

Tominaga O, Su ZH, Kim CG, Okamoto M, Imura Y, Osawa S: **Formation of the Japanese carabina fauna inferred from a phylogenetic tree of mitochondrial ND5 gene sequences (Coleoptera, Carabidae).** *J Mol Evol* 2000, **50:**541-549.

Whiting MF: **Mecoptera is paraphyletic: multiple genes and phylogeny of Mecoptera and Siphonaptera.** *Zool Scr* 2002, **31:**93-104.
